# Normative data for the self-reported and parent-reported Strengths and Difficulties Questionnaire (SDQ) for ages 12–17

**DOI:** 10.1186/s13034-021-00437-8

**Published:** 2022-01-18

**Authors:** Jorien Vugteveen, Annelies de Bildt, Marieke E. Timmerman

**Affiliations:** 1 Heymans Institute for Psychological Research, University of Groningen, Groningen, The Netherlands; 2 Hanze University of Applied Sciences, Groningen, The Netherlands; 3 Department of Psychiatry, Child and Adolescent Psychiatry, University Medical Center Groningen (UMCG), University of Groningen, Groningen, The Netherlands; 4 Accare Child and Adolescent Psychiatry, Groningen, The Netherlands

**Keywords:** Normative study, Multi-informant ratings, Mental health, Psychosocial functioning, Continuous regression-based norming, Gender differences, Age differences, CBCL, YSR

## Abstract

**Background:**

The Strengths and Difficulties Questionnaire (SDQ) is widely used to screen for psychosocial problems among adolescents. As the severity of such problems is known to be related to age and gender, screening could be improved by interpreting SDQ scale scores with age-specific and perhaps gender-specific norms. Up to now, such norms are lacking. The aim of the current study is to present gender-specific and joint normative data per year of age for the Dutch self-reported and parent-reported SDQ versions for use among 12- to 17-year-old adolescents.

**Methods:**

The norm groups for the self-reported and parent-reported SDQ versions consisted of 993 adolescents and 736 parents, respectively, from the general Dutch population. Per SDQ version, both gender-specific norms and joint norms (percentiles and cutoffs) per year of age were calculated through regression-based norming (Rigby in J Roy Stat Soc Ser C 54:507, 2005). Additionally, these norms were compared to the widely used British norms that are neither age-specific nor gender-specific.

**Results:**

By design, gender-specific ‘abnormal’ cutoffs (i.e., cutoffs aimed at identifying max. 10% of the most extremely scoring males and max. 10% of the most extremely scoring females) resulted in about equal percentages of ‘abnormal’ scoring male and female adolescents per SDQ scale. In contrast, joint ‘abnormal’ cutoffs (i.e., cutoffs aimed at identifying max. 10% of the most extremely scoring adolescents) resulted in relatively more male (7.6 to 13.6%, depending on age) than female (3.3 to 8.9%, depending on age) adolescents as scoring ‘abnormal’ on scales measuring externalizing behavior (self-reported and parent-reported SDQ versions), and relatively more female (3.9 to 14.3%, depending on age) than male (1.8 to 6.9%, depending on age) adolescents as scoring ‘abnormal’ on scales measuring internalizing behavior (self-reported SDQ version). In both types of norms, minor age effects were present. Among Dutch adolescents, the British norms yielded detection rates much lower than the expected 10%.

**Conclusions:**

Our findings indicate that detection rates depend on the reference group that is used (British or Dutch general adolescent population; specific gender group or not). The normative data in this paper facilitate the comparison of an adolescent’s scores to different reference groups, and allow for cross-country/cultural comparisons of adolescents’ psychosocial behavior.

**Supplementary Information:**

The online version contains supplementary material available at 10.1186/s13034-021-00437-8.

## Background

The Strengths and Difficulties Questionnaire (SDQ) [16, 17, 19] is widely used to screen for psychosocial problems among adolescents. The questionnaire is valued for several reasons, among which the availability of SDQ versions for adolescents themselves, their parent(s) and teacher(s), and its focus on both strengths and difficulties, whereas many other questionnaires only focus on problems. The SDQ covers five domains of psychosocial behaviour: emotional difficulties, conduct difficulties, hyperactivity/inattention, social difficulties and prosocial behaviour. The questionnaire is relatively short, especially in comparison to the well-known Child Behavior Checklist (CBCL [1]) and its self-report version the Youth Self Report (YSR [2]) that contain scales measuring similar concepts.

An individual’s SDQ scale scores are typically interpreted using norms based on the general population. For the SDQ, cutoffs based on these norms are typically determined so that the scores of the 10% most extremely scoring individuals (i.e., high on the difficulties scales, low on the prosocial behavior scale) are classified as ‘abnormal’, the scores of the 10% next-to-most-extremely scoring individuals as ‘borderline’, and the rest as ‘normal’ [16]. Thus, the classifications are based on norms corresponding with the 80th and 90th percentiles for the difficulties scales, and the 10th and 20th percentiles for the prosocial behavior scale.

Since its development, norms were published for the original English SDQ and for several translations of its self-reported and parent-reported versions. The use of these versions is supported by ample evidence for their validity for screening purposes [6, 18, 34, 44, 45, 51]. To gain understanding of how useful the norms for these SDQ versions are among adolescents, three aspects are important to consider. The first is the availability of age-specific norms. As severity of psychosocial problems is known to be related to age [10, 12], norms for adolescents should be calculated based on a sample consisting of only adolescents. We found such norms for multiple parent-reported SDQ translations (e.g., Danish [5], Dutch [24], English, USA [20], English, Australia [26], Italian [42], Japanese [29], Swedish [7], and several self-reported SDQ translations (e.g., Danish [5], English, UK [19], Hebrew [23]). Only the norms for the Swedish parent-reported version include norms per year of age (10 to 13 years). These norms show that SDQ scale scores correspond to different percentile ranks across age groups. This suggests that norms per year of age are more appropriate than norms covering larger age ranges.

The second aspect to consider is the national or geographical background of the individuals in the adolescent sample that the norms were based on. For both the parent-reported [5, 7, 24, 42] and the self-reported [5, 19] SDQ versions, the SDQ scale score identified as cutoff for the ‘abnormal’ classification (90th percentile) differed somewhat across language versions, suggesting that norms are potentially of limited use within national, cultural or geographical populations other than the population the norms were determined for.

The third aspect to consider is whether the available norms are gender-specific or not. Gender-specific norms allow for comparing an adolescent’s scores to the scores of other adolescents of the same gender. Applying the ‘abnormal’ cutoffs based on these norms results in identification of the 10% most extremely scoring adolescents per gender group. In contrast, joint norms allow for comparing an adolescent’s scores to those of adolescents in general. Applying the ‘abnormal’ cutoffs based on these norms results in identification of the 10% most extremely scoring adolescents, thereby potentially identifying relatively more males than females for some subscales, and vice versa for others. The preference for either gender-specific or joint norms depends on whether SDQ scales measure the intended strengths and difficulties in the same way among male and female adolescents (i.e., whether measurement invariance holds across gender). Joint norms are more appropriate if measurement invariance holds, and gender-specific norms are if it does not. Note that even when a measurement invariance analysis [28] would yield no evidence against measurement invariance, measurement invariance cannot be ruled out. If all items within a scale have a different meaning for boys than for girls, there is no way to distinguish between lack of measurement invariance and difference in means of latent scores across genders. Underlying this gender-specific versus joint norm preference is a debate about (a) to what extent the DSM-IV [4] and ICD-10 [54] criteria on which the SDQ items were based, are valid for both genders (e.g., SDQ scales were found to be predictive for Attention-Deficit/Hyperactivity Disorder (ADHD) and Autism Spectrum Disorder (ASD) e.g. [6, 16, 49]; for both disorders, the criteria have been identified as being based on male representations of the disorders [3, 13, 30, 52]), (b) how stereotypes affect the accuracy of recognizing and reporting an adolescent’s problem behavior by individuals who are key to referral and diagnostic processes, and (c) who needs to be identified with the help of SDQ scale scores (e.g., do we want to identify adolescents who manage to compensate for their symptoms or not?).

For Dutch adolescents, norms based on an adolescent sample are available for the parent-reported SDQ version only [24]. These norms are neither age-specific nor gender-specific, and they have two additional weaknesses. The first is that the accuracy of these norms may be affected, because the normative sample was potentially not fully representative of the Dutch adolescent population and relatively small (*n* = 395). Consequently, the resulting cut-off scores may be based on biased norm estimates with substantial uncertainty due to sampling fluctuations. The second weakness of these norms is that they only include norm scores approximately corresponding with the 90th percentile, therewith identifying the ‘abnormal’ category; norms for other categories are lacking. This dichotomization of SDQ scores implies a loss of information, and is arguably less useful for mental healthcare professionals. To better facilitate them, it would be useful to (re)determine norms for the Dutch adolescent-reported and parent-reported SDQ versions. The use of these two versions is supported by ample evidence for their construct validity, including convergent and discriminative [40, 45, 50, 51], and criterion validity [40, 49, 51].

The aim of the current study is to present gender-specific and joint normative data per year of age for the self-reported and parent-reported SDQ versions for use among 12- to 17-year-old Dutch adolescents. We aim to present accurate norms for the Dutch general adolescent population by calculating norms using data from adolescent samples of decent sizes (self-report version: *n* = 993; parent-report version: *n* = 736), while accounting for potential sample representativity problems regarding gender, socioeconomic status, and ethnic background. Additionally, the data were interpreted with the widely used British norms and with the published but potentially moderately useful Dutch norms, that are both neither age-specific nor gender-specific.

## Methods

### Norm groups

SDQ data were collected in three waves at schools for secondary education: (1) in 2009/2010 data were collected from 519 13- to 14-year-old adolescents and their parents (estimated response rate: 50–60%), (2) between 2011 and 2013 from 331 12- to 17-year-olds and their parents (estimated response rate: 70%, see for a more detailed description), and (3) in 2016/2017 from 443 similarly aged adolescents and parents (estimated response rate: 80%). The data from wave one were gathered as part of a large study on the value of screening instruments in Dutch youth mental healthcare [47]. The data from wave two were gathered as part of a prospective cohort study aimed at obtaining evidence on the care chain for Dutch children and adolescents with psychosocial problems [46]. The data from wave three were gathered as part of a norming study of an intelligence test [20]. Respondents were recruited face-to-face or by phone. Informed consent was obtained from all participants’ parents. In case the participants were sixteen years or older, informed consent for participation was obtained from both the participant and their parent.

For 246 of the 1293 adolescents and parents, information was missing on their age, ethnicity (as indicated by the mother’s native country), gender, and/or socioeconomic status (as indicated by the mother’s highest completed level of education). They were excluded from the analyses, as this information was crucial for checking the representativity of the sample. The remaining 1047 adolescents and parents form the norm groups for the self-reported (*n* = 993) and the parent-reported (*n* = 736) SDQ versions. Table 1 provides demographic information on these norm groups and, for comparison, on the Dutch population.

**Table 1:** Demographic characteristic of the adolescents with available SDQ self-reported data (n = 993), with available SDQ parent-reported data (n = 736), and the Dutch population

Characteristics	SDQ informant version		Dutch population^a^
	Self-report	Parent	
	*N* (%)	*N* (%^a^)	%
Gender			
Male	466 (46.9)	345 (46.9)	49.5
Female	527 (53.1)	391 (53.1)	50.5
Age			
12	82 (8.3)	68 (9.2)	16.5
13	253 (25.5)	186 (25.3)	16.3
14	249 (25.1)	190 (25.8)	16.4
15	151 (15.2)	127 (17.3)	16.9
16	151 (15.2)	97 (13.2)	16.9
17	107 (10.8)	68 (9.2)	17.1
Native country mother			
The Netherlands	885 (89.1)	668 (90.8)	78.6
Other	108 (10.9)	68 (9.2)	21.4
Educational level mother			
Low	238 (24.0)	163 (22.1)	23.6
Medium	411 (41.4)	320 (43.5)	41.7
High	344 (34.6)	253 (34.4)	34.7

*SDQ* Strengths and Difficulties Questionnaire

^a^the information on the distributions in the Dutch population was retrieved from Statistics Netherlands [39]

### The Strengths and Difficulties Questionnaire

Adolescents and their parents completed official Dutch translations [45] of the self-reported and parent-reported SDQ, respectively. The 25 items of both versions are evenly divided over five scales: one focusing on strengths (prosocial behavior) and four scales focusing on difficulties (emotional, conduct, hyperactivity, and social problems). All difficulties items together form the total difficulties scale [16, 17]. Additionally, the conduct problems and hyperactivity/inattention items together form the externalizing difficulties scale, and the emotional and peer problem items together form the internalizing difficulties scale [15]. All items are rated on a three-point scale (0 = *not true*, 1 = *somewhat true*, and 2 = *certainly true*). Five positively worded items belonging to different SDQ difficulties scales are reverse-coded. High scores on the difficulties scales represent a high degree of difficulties; a high score on the prosocial scale represents a high degree of prosocial behavior.

### Statistical analysis

#### Descriptive statistics and previously existing norms

To characterize the samples of adolescents (males, females, total), mean scale scores and standard deviations were calculated per gender group and without distinguishing between genders. Additionally, we calculated the percentages of adolescents (males, females, total) that were identified as scoring in the ‘abnormal’ range, using previously existing cutoffs. That is, the internationally widely used United Kingdom (UK) [19] cutoffs were applied to the self-reported SDQ scale scores; Dutch [24] and UK [16] cutoffs were applied to the parent-reported SDQ scale scores. Note that the UK cutoffs for the parent-reported version were determined based on a UK sample consisting of both children and adolescents. Because they are not age-specific and from a different country they may be of limited use among adolescents. The Dutch norms for the SDQ parent-reported version were only recently established and such norms for the SDQ self-reported version are still lacking, therefore these UK norms were widely used in the Dutch general adolescent population, and still are.

#### Norm group representativity

To optimize the accuracy of the SDQ norms that were established, we first ensured the representativity of the norm groups for the different reference populations (adolescents in general, males, and females). This was done by checking and, if necessary, correcting for deviations from the Dutch population regarding the distributions of gender, ethnic background (as indicated by the mother’s native country) and socioeconomic status (as indicated by the mother’s highest completed educational level). Note that possible deviations of the norm group distributions regarding age are irrelevant, because we computed age-specific norms. The information in Table 1 indicates that the norm groups for both SDQ versions were not fully representative of the Dutch population of adolescents regarding gender and ethnic background (no problems were detected for socioeconomic status), with an overrepresentation of females and adolescents with a Dutch background. For calculating the joint norms (i.e., without distinguishing between gender groups), the deviations were corrected for by weighing on ethnic background and gender. For calculating the gender-specific norms, the correction was performed by weighing on ethnic background. The weights used and information on how these weights were established are presented in Additional file 1.

#### Establishing norms

Norms were determined through regression-based norming performed in R [32], using generalized additive models for location, scale, and shape (GAMLSS) [37], following the strategy as outlined in [41]. Regression-based norming allows us to estimate the population distribution of scores per SDQ scale as a continuous function of age (i.e., without splitting up our norm groups into subgroups with certain intervals of age). We opted for this approach because it allows all data to be used simultaneously to establish norms, instead of norms being calculated separately for each subgroup that may or may not be large enough to sensibly perform the necessary calculations on.

Per SDQ version (adolescent, parent), gender-specific norms and joint norms were calculated for 8 scales (1 strengths scale, 4 difficulties scales, 1 total difficulties scale, 1 externalizing difficulties scale, 1 internalizing difficulties scale). Thus, the analyses were performed for each of the SDQ scales separately, yielding 8 (scales) × 2 (versions) × 3 (types: norms for females, norms for males, joint norms) = 48 norms. Possible scores on the total difficulties scale range from 0 to 40, which can be approximated with a continuous distribution. The population distribution for this scale was estimated using the Box-Cox power exponential (BCPE) distribution [35]. The BCPE distribution has four parameters: *µ* for the location of the distribution (median), *σ* for its scale (approximate coefficient of variation), *ν* for its skewness (degree of symmetry), and *τ* for its kurtosis (level of ‘peakedness’). The possible scores for the five strengths and difficulties scales (excluding the total difficulties scale) range from 0 to 10, and for the externalizing and internalizing difficulties scales from 0 to 20. These score distributions cannot be reasonably approximated with a continuous distribution. The population distributions for these scales were estimated using the beta binomial (BB) distribution for ordered categorical variables [36]. The BB distribution has two parameters: *µ* for the location of the distribution (mean) and *σ* for its scale (approximate coefficient of variation).

In order to calculate the joint norms per year of age (12 through 17), the regression models for all SDQ scales for both SDQ versions included age as predictor for the population distribution parameters (i.e., *µ*, *σ* for all scales, and also *ν, τ* for the total difficulties scale). To consider both linear and more complex associations between age and the distribution parameters, age was included using polynomials. We considered models including polynomials up to degree 6 (i.e., age^1^, age^2^, …, age^6^) for each distribution parameter. Per SDQ scale of both SDQ versions (i.e., 16 scales in total), the model with the polynomials resulting in the smallest Bayes Information Criterion (BIC) [38] value was selected. Their fit to empirical data was assessed through visual inspection of worm plots [8]; if needed the models would have been adapted, but the resulting plots did not warrant any further actions. The selected models were used to calculate the norms per year of age.

For calculating gender-specific norms, the regression models included both age and gender as predictors for the parameters. Age was included using polynomials, in the same way as for the joint norms. Gender was included as factor as it had two possible values (male, female). Models including the interaction between age and gender were considered. For each of the 16 estimated SDQ scales, the estimated model resulting in the smallest BIC value was selected for visual inspection of its fit, and used (if needed after adaptation) for calculating the gender-specific norms per year of age.

Based on the calculated norms, we established cutoffs. That is, we identified ‘abnormal’ SDQ cutoffs to indicate *up to* 10% of the most extremely scoring adolescents (10th percentile for the prosocial behavior scale and 90th percentile for all other scales), and the ‘borderline’ cutoffs to indicate *up to* 10% of the next-to-most-extremely scoring adolescents (20th percentile for the prosocial behavior scale and 80th percentile for all other scales). This approach is in line with how the American (USA) [21], the Australian [26], and the Danish [5] norms were determined. In contrast, the Chinese [11], the pre-existing Dutch [24], and the Japanese [29] norms were determined to identify *approximately* the percentages mentioned above. For the other adolescent norms, we were unable to determine with certainty which of the two approaches were used. Note that the normative data provided in this paper and its corresponding additional files can easily be used to establish cutoffs aimed at *approximately* identifying ten and twenty percent (or any other percentage) of extremely scoring adolescents.

#### Scale reliabilities

Per SDQ scale, the reliability of the observed scores was computed using the nonlinear structural equation modelling reliability coefficient [55], based on a one-factor confirmatory factor analysis model with equal item loading per factor (tau equivalence). This type of reliability coefficient is suitable in this context, because it takes into account the ordinal nature of the SDQ items. For the purpose of comparability with other studies, Cronbach’s alpha coefficients computed as well. The analyses were performed in R [32], using the semTools package [22].

In contrast to the regression-based norming procedures, the reliability analyses were performed at item level. At this level, our dataset contained some missing data for the self-reported (M = 0.20%, SD = 0.27, min = 0.0%, max = 1.1%) and the parent-reported (M = 0.2%, SD = 0.18, min = 0.0%, max = 0.7%) SDQ versions. We used single two-way imputation with normally distributed errors to impute the missing data [43].

## Results

### SDQ scale scores

Table 2 presents mean self-reported and parent-reported scale scores and standard deviations per gender group (males, females) and without distinguishing between genders. The mean self-reported total difficulties scale scores were 8.7 (*sd* = 5.1) for females, 8.5 (*sd* = 4.9) for males, and 8.6 (*sd* = 5.0) without distinguishing between genders. The mean parent-reported total difficulties scale scores were 6.3 (*sd* = 5.3) for females, 7.8 (*sd* = 5.8) for males, and 7.0 (*sd* = 5.6) without distinguishing between genders. Note that the *sd* values are mainly provided for comparability with other studies. The values provide limited information about the amount of variation in the data as the data do not approximate a normal distribution. Table 3 presents reliability estimates per scale of the self-reported and parent-reported SDQ version.

**Table 2:** Per SDQ version (self-reported, parent-reported), mean scale scores and standard deviations for male and female adolescents

SDQ version	SDQ scale	Gender group								
		Females	% ‘abnormal’ ^a^		Males	% ‘abnormal’		Total	% ‘abnormal’	
		*M (SD)*	UK ^b^	Dutch ^c^	*M (SD)*	UK	Dutch	*M (SD)*	UK	Dutch
Self-reported	Emotional	2.8 (2.3)	96.4	–	1.7 (1.8)	99.6	–	2.3 (2.1)	97.9	–
	Conduct	1.2 (1.1)	99.4	–	1.5 (1.4)	98.7	–	1.3 (1.3)	99.1	–
	Hyperactivity	3.5 (2.4)	92.8	–	3.8 (2.4)	91.4	–	3.7 (2.4)	92.1	–
	Social	1.3 (1.5)	98.5	–	1.4 (1.6)	99.4	–	1.4 (1.6)	99.6	–
	Prosocial	8.4 (1.4)	99.1	–	7.7 (1.7)	95.7	–	8.1 (1.6)	97.5	–
	Externalizing	4.7 (3.1)	–	–	5.4 (3.2)	–	–	5.0 (3.1)	–	–
	Internalizing	4.1 (3.2)	–	–	3.1 (2.8)	–	–	3.6 (3.1)	–	–
	Total	8.7 (5.1)	97.2	–	8.5 (4.9)	97.6	–	8.6 (5.0)	97.4	–
Parent-reported	Emotional	2.0 (2.1)	93.6	89.3	1.7 (2.1)	93.0	87.8	1.9 (2.1)	93.3	88.6
	Conduct	0.9 (1.2)	98.2	90.0	1.0 (1.5)	96.2	87.0	1.0 (1.4)	97.3	88.6
	Hyperactivity	2.1 (2.3)	91.3	93.6	3.3 (2.6)	96.7	86.4	2.7 (2.5)	94.2	90.2
	Social	1.3 (1.7)	94.1	94.1	1.7 (1.8)	90.1	90.1	1.5 (1.8)	92.3	92.3
	Prosocial	8.5 (1.8)	95.7	86.4	8.1 (1.9)	93.9	81.7	8.3 (1.8)	94.8	84.2
	Externalizing	3.0 (3.0)	–	94.4	4.4 (3.5)	–	87.5	3.7 (3.3)	–	91.2
	Internalizing	3.3 (3.4)	–	90.5	3.4 (3.3)	–	87.5	3.4 (3.3)	–	89.1
	Total	6.3 (5.3)	94.6	92.1	7.8 (5.8)	93.0	87.0	7.0 (5.6)	93.9	89.7

*SDQ* Strengths and Difficulties Questionnaire

^a^The percentage of adolescents with SDQ scores that are considered ‘abnormal’ using ^b^ the UK [16, 19] cutoffs (not available for the externalizing and internalizing difficulties scales) and ^c^ the Dutch [24] cutoffs (not available for the SDQ self-reported version)

**Table 3:** Per SDQ version, Cronbach’s alpha (α) and nonlinear structural equation modelling reliability (ρ_NL_) coefficients

SDQ scale	*α*	ρ_NL_	*α*	ρ_NL_
	Self-reported SDQ version		Parent-reported version	
Emotional	0.71	0.73	0.75	0.84
Conduct	0.47	0.51	0.57	0.63
Hyperactivity	0.76	0.79	0.78	0.87
Social	0.56	0.59	0.61	0.65
Prosocial	0.58	0.61	0.72	0.75
Externalizing	0.74	0.81	0.77	0.90
Internalizing	0.72	0.79	0.77	0.88
Total	0.69	0.76	0.74	0.86

### Applying pre-existing UK and Dutch general population norms

Table 2 further shows per SDQ scale what percentage of adolescents (males, females, total) is identified as scoring in the ‘abnormal’ range, using pre-existing UK and Dutch cutoffs. The results indicated cutoffs based on the UK norms to yield detection rates much lower than the intended 10% of the most extremely scoring adolescents. The cutoffs based on the previously existing Dutch norms for the parent-reported SDQ version yielded varying results, with detection rates close to 10% for some scales and much lower or higher for other scales. Note that these pre-existing norms are neither age-specific nor gender-specific.

### Establishing gender-specific and age-specific norms

The complete norms for all age and gender groups can be found in Tables S1–S6 (Additional file 2). As these tables contain an abundance of information that cannot easily be described succinctly, we here present norms only for 15-year-old male and female adolescents for all scales of the parent-reported SDQ version. For all other combinations of age (12 to 17, six ages in total), gender (female, male, total) and SDQ version (adolescent, parent) we present only ‘borderline’ and ‘abnormal’ cutoff values.

Table 4 presents the norms for 15-year-old male and female adolescents for all eight scales of the parent-reported SDQ version, as an example of what the norms look like. Within the 15-year-old age group and for the parent-reported SDQ version, the norms show higher occurrence rates of hyperactivity/inattention and externalizing problems for male than for female adolescents. Consequently, the cutoff values for classifying scores on these scale as ‘borderline’ or ‘abnormal’ are higher for males than for females. For example, for females hyperactivity scale scores ≥ 5 are considered ‘abnormal’, whereas for males scores ≥ 7 are considered as such.

**Table 4:** Percentiles for the parent-reported SDQ version for 15-year-old males and females

SDQ scale	Gender	SDQ scale score											
		0	1	2	3	4	5	6	7	8	9	10	11
Emotional	F	31.9	52.9	68.1	79.0	86.9	92.3	95.9	98.1	99.3	99.8	100	
	M	39.0	58.2	71.0	80.1	86.8	91.7	95.1	97.5	98.9	99.7	100	
Conduct	F	49.8	75.8	89.1	95.4	98.2	99.4	99.8	100	100	100	100	
	M	52.3	73.3	84.9	91.7	95.6	97.9	99.1	99.7	99.9	100	100	
Hyper	F	31.8	52.3	67.1	77.9	85.8	91.5	95.3	97.7	99.1	99.8	100	
	M	14.5	30.7	46.3	60.3	72.2	81.8	89.2	94.4	97.6	99.4	100	
Social	F	43.1	65.2	78.9	87.6	93.1	96.4	98.3	99.3	99.8	100	100	
	M	28.8	52.4	69.8	82.0	90.0	94.9	97.7	99.1	99.7	100	100	
Prosocial	F	100	99.8	99.2	98.0	95.6	91.4	84.5	73.8	57.8	34.4	0.0	
	M	100	99.9	99.5	98.3	95.6	90.2	80.8	66.1	45.6	21.3	0.0	
Externalizing	F	23.2	41.3	55.5	66.7	75.5	82.2	87.3	91.2	94.0	96.0	97.5	98.4
	M	12.1	25.7	38.8	50.7	61.1	69.9	77.3	83.2	87.9	91.6	94.3	96.3
Internalizing	F	20.0	36.6	50.3	61.5	70.6	77.9	83.7	88.2	91.6	94.2	96.1	97.5
	M	17.5	33.0	46.3	57.6	66.9	74.7	80.9	85.9	89.8	92.8	95.1	96.8
Total	F	0.1	11.5	21.7	32.0	42.1	51.4	59.6	66.4	72.1	76.8	80.7	83.9
	M	0.0	7.8	15.1	22.9	31.0	39.1	47.0	54.4	60.9	66.6	71.6	75.8

SDQ scale	Gender	SDQ scale score											
		11	12	13	14	15	16	17	18	19	20	30	40
	M												
Emotional	F												
	M												
Conduct	F												
	M												
Hyper	F												
	M												
Social	F												
	M												
Prosocial	F												
	M	98.4	99.1	99.5	99.7	99.9	99.9	100	100	100	100		
Externalizing	F	96.3	97.7	98.6	99.3	99.6	99.8	99.9	100	100	100		
	M	97.5	98.5	99.1	99.5	99.8	99.9	100	100	100	100		
Internalizing	F	96.8	97.9	98.8	99.3	99.6	99.8	99.9	100	100	100		
	M	83.9	86.6	88.8	90.6	92.1	93.4	94.4	95.3	96.0	96.7	99.3	100
Total	F	75.8	79.4	82.4	85.1	87.3	89.2	90.8	92.1	93.3	94.3	98.8	100

*SDQ* Strengths and Difficulties Questionnaire, *F* females, *M* males

Norms for all other age groups per SDQ version (self-reported, parent-reported) are presented in Tables S1–S6 (Additional file 2)

### Joint and gender-specific norms

Tables 5 and 6 present the gender-specific and joint cutoff values per year of age for the self-reported and parent-reported SDQ versions, respectively. To gain insight into the main differences between the gender-specific norms and the joint norms, we applied the ‘abnormal’ cutoffs based on both types of norms to the scores of all adolescents in our norm groups. The gender-specific ‘abnormal’ cutoffs were established to identify a maximum of 10% of adolescents per gender group, resulting in identification of fairly equal percentages of male and female adolescents as scoring ‘abnormal’. In contrast, the joint ‘abnormal’ cutoffs were established to identify a maximum of 10% of all adolescents, resulting in the identification of relatively more male than female adolescents as scoring ‘abnormal’ on scales measuring externalizing problems (self-reported and parent-reported SDQ versions), and of relatively more female than male adolescents as scoring ‘abnormal’ on scales measuring internalizing problems (self-reported SDQ version). Below these gender differences are described in more detail. The percentages presented can be verified using the cutoffs presented in Tables 5 and 6 in combination with the information in Additional file 2: Tables S1, S2, S4, and S5.

**Table 5:** Cutoff values per year of age for the SDQ self-report version for females, males, and without distinguishing between genders

Age	SDQ scale															
	Emotional		Conduct		Hyperactivity		Social		Prosocial		Externalizing		Internalizing		Total	
	‘Bord.’	‘Abn.’	‘Bord.’	‘Abn.’	‘Bord.’	‘Abn.’	‘Bord.’	‘Abn.’	‘Bord.’	‘Abn.’	‘Bord.’	‘Abn.’	‘Bord.’	‘Abn.’	‘Bord.’	‘Abn.’
Females																
12	4	6	2	3	6	7	2	3	−^a^	6	7	9	6	8	13	16
13	5	6	2	3	6	7	2	3	6	5	7	9	6	9	13	16
14	5	6	2	3	6	7	2	3	6	5	7	9	7	9	13	16
15	5	6	2	3	6	7	2	3	6	5	7	9	7	9	13	16
16	5	6	2	3	6	7	2	3	6	5	7	9	7	9	13	17
17	5	6	2	3	6	7	2	3	6	5	7	9	7	9	13	17
Males																
12	3	4	3	4	6	7	3	4	5	4	8	10	5	7	13	16
13	3	4	3	4	6	7	3	4	5	4	8	10	5	7	13	16
14	3	4	3	4	6	7	3	4	5	4	8	10	5	7	13	16
15	3	4	−^a^	3	6	7	3	4	5	4	8	10	6	7	13	16
16	3	4	−^a^	3	6	7	3	4	5	4	8	10	6	7	13	16
17	4	5	2	3	6	7	3	4	5	4	8	10	6	7	13	16
Total																
12	4	5	−^a^	3	6	7	3	4	6	5	8	10	6	8	13	16
13	4	5	2	3	6	7	3	4	6	5	8	9	6	8	13	16
14	4	5	2	3	6	7	3	4	6	5	8	9	6	8	13	16
15	4	5	2	3	6	7	3	4	6	5	8	9	6	8	13	16
16	4	6	2	3	6	7	3	4	6	5	8	9	6	8	13	16
17	5	6	2	3	6	7	3	4	6	5	8	9	6	8	13	16

*SDQ* Strengths and Difficulties Questionnaire, *‘Bord.’* ‘borderline’, *‘Abn.’*‘abnormal’

For all SDQ scales except the prosocial scale, scale scores equal to or higher than the displayed cutoff value (≥ 90th percentile) are considered ‘abnormal’. For the prosocial scale, scale scores equal to or lower than the displayed cutoff value (≤ 10th percentile) are considered ‘abnormal’

For all SDQ scales except the prosocial scale, scale scores equal to the displayed cutoff value up to the cutoff value for ‘abnormal’ (80th up to the 90th percentile) are considered ‘abnormal’. For the prosocial scale, scale scores scale scores equal to or lower than the displayed cutoff value (20th down to the 10th percentile) are considered ‘borderline’

^a^Borderline cutoff score unavailable due to right skewed score distribution and the limited number of possible scale scores, resulting in large jumps in percentile ranks and skipping the 80th up to the 90th percentiles

The complete norms for all ages and scales of the self-reported SDQ version are presented in Tables S1–S3 (Additional file 2)

**Table 6:** Cutoff values per year of age for the SDQ parent-report version for females, males, and without distinguishing between genders

Age	SDQ scale															
	Emotional		Conduct		Hyperactivity		Social		Prosocial		Externalizing		Internalizing		Total	
	‘Bord.’	‘Abn.’	‘Bord.’	‘Abn.’	‘Bord.’	‘Abn.’	‘Bord.’	‘Abn.’	‘Bord.’	‘Abn.’	‘Bord.’	‘Abn.’	‘Bord.’	‘Abn.’	‘Bord.’	‘Abn.’
Female																
12	4	5	2	3	5	6	2	4	6	5	6	8	6	8	11	15
13	4	5	2	3	4	6	3	4	6	5	6	8	6	8	11	15
14	4	5	2	3	4	6	3	4	6	5	5	7	6	8	11	14
15	4	5	2	3	4	5	3	4	6	5	5	7	6	8	10	14
16	4	5	2	3	3	5	3	4	6	5	5	7	6	8	10	14
17	4	5	2	3	3	5	3	4	6	5	5	7	6	8	10	13
Males																
12	4	5	2	3	6	8	3	5	6	5	8	10	6	9	13	18
13	4	5	2	3	6	7	3	5	6	5	8	10	6	9	13	18
14	4	5	2	3	6	7	3	5	6	5	7	9	6	9	13	17
15	3	5	2	3	5	7	3	4	6	5	7	9	6	9	13	17
16	3	5	2	3	5	6	3	4	5	4	7	9	6	8	12	17
17	3	5	2	3	5	6	3	4	5	4	6	8	6	8	12	16
Total																
12	4	5	2	3	6	7	3	4	6	5	6	10	6	9	13	17
13	4	5	2	3	5	7	3	4	6	5	6	9	6	8	12	17
14	4	5	2	3	5	6	3	4	6	5	6	9	6	8	12	16
15	4	5	2	3	5	6	3	4	6	5	6	8	6	8	11	16
16	3	5	2	3	4	6	3	4	6	5	6	8	6	8	11	15
17	3	5	2	3	4	5	3	4	6	5	5	7	6	8	11	14

*SDQ* Strengths and Difficulties Questionnaire, *‘Bord.’* ‘borderline’, *‘Abn.’* ‘abnormal’

For all SDQ scales except the prosocial scale, scale scores equal to or higher than the displayed cutoff value (≥ 90th percentile) are considered ‘abnormal’. For the prosocial scale, scale scores equal to or lower than the displayed cutoff value (≤ 10th percentile) are considered ‘abnormal’

For all SDQ scales except the prosocial scale, scale scores equal to the displayed cutoff value up to the cutoff value for ‘abnormal’ (80th up to the 90th percentile) are considered ‘abnormal’. For the prosocial scale, scale scores scale scores equal to or lower than the displayed cutoff value (20th down to the 10th percentile) are considered ‘borderline’

The complete norms for all ages and scales of the parent-reported SDQ version are presented in Tables S4–S6 (Additional file 2)

### Externalizing problems

For the *self-reported* SDQ version, applying the joint ‘abnormal’ cutoffs resulted in the identification of 10.5% (7.3 to 11.3%, depending on the adolescent’s age) of males and 7.7% (5.2 to 8.3%) of females as scoring ‘abnormal’ on the externalizing difficulties scale. Further, 9.8% (7.6 to 12.1%) of males and 4.4% (3.3 to 5.7%) of females were identified as scoring ‘abnormal’ on the conduct difficulties scale, and 7.5% (6.6 to 8.4%) of males and 6.6% (5.8 to 7.5%) of females were identified as doing so on the hyperactivity difficulties scale. For the *parent-reported* SDQ version, applying the joint ‘abnormal’ cutoffs resulted in the identification of 11.0% (8.9 to 13.6%) of males and 5.4% (4.2 to 6.9%) of females as scoring ‘abnormal’ on the externalizing difficulties scale. Further, 8.2% (7.6 to 8.7%) of males and 4.4% (4.1 to 5.0%) of females were identified as scoring ‘abnormal’ on the conduct difficulties scale, and 11.0% (8.8 to 13.2%) of males and 4.7% (3.7 to 5.9%) of females were identified as doing so on the hyperactivity difficulties scale.

### Internalizing problems

For the *self-reported* SDQ version, applying the joint ‘abnormal’ cutoffs resulted in the identification of 5.9% (5.8 to 6.1%) of males compared to 10.7% (10.0 to 11.7%) of females as scoring ‘abnormal’ on the internalizing difficulties scale. Further, 3.3% (1.8 to 4.2%) of males and 11.6% (8.3 to 14.3%) of females were identified as scoring ‘abnormal’ on the emotional difficulties scale, and 6.2% (5.6 to 6.9%) of males and 4.5% (3.9 to 5.1%) of females were identified as doing so on the social difficulties scale. For the *parent-reported* SDQ version, no substantial gender differences in reported internalizing problems were found.

## Discussion

The SDQ is widely used to screen for psychosocial problems among adolescents. Norms for interpreting SDQ scale scores are available for multiple language versions of the questionnaire. However, for none of these language versions joint norms and gender-specific norms per year of age were established, even though the occurrence of psychosocial problems is known to be related to age [10, 12] and gender [9, 27, 48]. We addressed this issue by providing such norms for the Dutch self-reported and parent-reported SDQ versions for use among 12- to 17-year-old adolescents. The norms showed the presence of age- and gender-effects in the reported extent to which problems occur.

The Dutch self-reported and parent-reported SDQ versions were introduced in 2003 [45], with UK joint norms available for interpreting SDQ scale scores [16, 19]. In 2019, Dutch norms were provided for the parent-reported SDQ version [24]. In our norm groups, we found cutoffs based on the UK norms and the pre-existing Dutch norms to yield detection rates substantially different from the intended 10% of the most extremely scoring adolescents. Compared to the pre-existing UK and Dutch norms, we presume our newly established norms to be more useful for interpreting Dutch adolescents’ scores because they are (a) fairly recent (norms can become outdated [14, 53]), (b) age-specific, (c) available for the self-reported and the parent-reported SDQ versions, (d) established using regression-based (i.e., continuous) norming, and (e) based on decent sample sizes, with representativity issues corrected for. Besides, we provide not only joint norms, but also gender-specific norms, therewith facilitating comparison of an adolescent’s scores to different reference groups.

The norms that we provide are so-called relative norms, and the resulting norm-referenced test scores express the relative position of the adolescent in comparison to his peers [25]. This relative norming approach is common in screening practice, as scores of individuals are typically interpreted relative to problem occurrence rates in a community population. A different approach is the criterion-referenced approach, where the criterion-referenced test score expresses the position of an adolescent in relation to an external criterion or standard [25]. This approach has been applied to obtain norms for the total difficulties scale of the self-reported SDQ version, with student’s subjective school well-being scores as the criterion [33]. The criterion-referenced approach is preferred over the relative approach when a clear, univocal external criterion is present for a test, and that criterion can be measured reliably. In the absence of such a clear ‘golden’ criterion, the relative approach is preferred, because of its clear interpretation, i.e. where the adolescent stands in relation to his peers.

In this paper, reliability estimates for the SDQ scales were presented. These estimates suggested that the conduct and peer difficulties scales of both the self-reported and the parent-reported SDQ versions as well as for the prosocial behaviour scale of the self-reported SDQ version are insufficiently reliable to warrant their use. While we acknowledge that these scales should be interpreted with some caution, we would also like to point out that criterion validity evidence was found for the conduct difficulties scale of both SDQ versions and the parent-reported social difficulties scale [49, 51]. These findings suggest that these scales are useful for screening purposes. The contradiction between some of the reliability findings and criterion validity findings possibly indicates that the scales in question measure sufficiently accurate in cases where it matters: among adolescents with more severe problems but not among adolescents without such problems. Investigating this issue goes beyond the scope of this paper, but it should be further examined, possibly using the test information function from the Item Response Theory (IRT) framework.

As a final note we would like to emphasize that the SDQ is not a diagnostic instrument. SDQ scores are meant to provide mental healthcare professionals with a preliminary indication of the nature and occurrence of problems an adolescent is experiencing. If the SDQ scores offer reason for concern, further clinical assessment is needed to determine how to best help the adolescent. Herewith it is important that clinicians are aware of the possible risk of stigmatizing an individual. These words of caution are further supported by the fact that we could not identify ‘borderline’ cutoff values for some scales of the self-reported SDQ version, because the scale scores were strongly skewed and the number of possible scores is rather limited. This shows that these scales in particular can only make a crude distinction between adolescents in terms of problem occurrence at the higher levels, and that further assessment of the individual child is needed to improve the understanding of the problems measured with these scales.

## Limitations

The validity of the norms presented in this paper is potentially affected by four aspects. The first is our effort to correct for norm group deviations from the Dutch adolescent population regarding ethnic background and gender by applying weights. To the best of our knowledge, this is an acceptable way to deal with these norm group representativity issues that presumably introduced little bias.

The second specifically regards the gender-specific norms. In the Dutch language, sex and gender are often indicated with the same word. As this word was used in the questionnaires, we do not know whether the resulting indications actually indicate gender. Taking into account the increased prevalence of adolescents with a gender identity that does not match their biological sex, calling our norms gender-specific might be somewhat inaccurate, as we cannot be sure that gender was provided for adolescents whose biological sex contrasts their gender identity.

The third aspect that potentially affects the validity of the norms in this paper is that the norm groups used to establish the norms resulted from combining three community samples, with the data from the most recent samples being gathered four to seven years after the data from the other samples were gathered. By handling these data as if it were one community sample, we assume that the occurrence rate of problems in the community population has not changed over time. We consider this assumption tenable, given the relatively short time span of maximally seven years between collecting the data of the three samples.

The fourth aspect that potentially affects the validity of the norms presented here is that our samples do not contain adolescents attending special education for lower cognitive levels (i.e. IQ lower than 55), for language, hearing, or vision impairments, or for behavioral problems. This means that adolescents with severe behavioural problems, who typically attend special education, are not represented in this study. We presume that the effect for representativity of the data is limited, because the parent and adolescent norm groups seem representative of their respective populations in terms of the socio-economic status, by proxy of the mother’s educational level.

Besides the self-reported and parent-reported SDQ versions, there is also a Dutch SDQ version available that can be completed by teachers. In this paper, we focus on the self-reported and parent-reported version, because the use of these two versions is supported by ample validity evidence [40, 44, 49–51]. Such information is not available for the Dutch teacher-reported version. A potential reason for the absence of such information is that teachers are less likely to be used as informants than adolescents themselves and their parents, because adolescents, compared to children, spend a very limited amount of time with each of their teachers. That being said, we do not know of existing evidence indicating that the teacher version should not be used during adolescence. Therefore, it could be useful to establish norms for the teacher-reported SDQ version as well.

In this paper, norms were established for 8 difficulties scale, but not for the impact scale that was later added to the SDQ [17]. If an adolescent experiences difficulties in any of the domains covered by the SDQ, the impact scale is meant to provide in indication of the chronicity, distress, and social impairment for the adolescent as well as burden for others. Consequently, we considered independently norming the impact scale irrelevant. Although beyond the scope of this paper, it would be useful to establish a method for interpreting impact scale scores in relation to scores on the difficulties scale of the Dutch self-reported and parents-reported SDQ versions.

## Conclusions

This study provides joint and gender-specific norms (percentiles) per year of age for all adolescent self-reported and parent rated Dutch SDQ scales, including the externalizing and internalizing difficulties scales. We provide percentiles for all possible scores of each SDQ scale, which allows for retrieving the ‘classic’ cutoffs (< 80th percentile = ‘normal’, 80–90 percentile = ‘borderline’, > 90th percentile = ‘abnormal’) as well as cutoffs corresponding to any other desired percentile. These normative data thus also allow for cross-country/cultural comparisons of adolescents’ psychosocial behavior.

The gender-specific norms yield different results than joint norms do. They confirm that females tend to report more internalizing problems and males and their parents tend to report more externalizing problems. The results show that detection rates depend on the reference group that is used to interpret SDQ scale scores provided by adolescents and their parents. Note that the results cannot be used to settle the debate on whether norms used in practice should be gender-specific or not. The latter question can be answered once agreement is reached in ongoing debate on, among other things, how valid the DSM-IV/ICD-10 criteria are for both genders and if/how stereotyping affects the processes for referral and diagnosing. Not knowing what the outcome of that debate will be, we present both types of norms, thereby facilitating the comparison of an adolescent’s scores to different reference groups: all similarly aged other adolescents or all similarly aged adolescents of the same gender.

In the Netherlands, an individual’s SDQ scale scores are typically interpreted using norms that were established decades ago based on a British sample. These norms are neither age-specific nor gender-specific. Our study shows that using those norms for interpreting SDQ scale scores provided by Dutch adolescents and their parents results in much lower detection rates than the intended 10% of the most extremely scoring adolescents. We strongly advice a reconsideration of using the British norms in Dutch (mental) healthcare practice.

## Supplementary Information

**Additional file 1:** (‘Additional file 1.pdf’) contains information on the weighting applied to correct over overrepresentation of females and adolescents with a Dutch background in the samples used to establish norms for the Dutch self-reported and parent-reported SDQ versions.

**Additional file 2:** (‘Additional file 2.pdf’) contains the complete norms (gender-specific and joint) per year of age for the self-reported and parent-reported SDQ versions.

## Data Availability

The Dutch SDQ versions are available from https://www.sdqinfo.com/. The data analyzed in the current study are not publicly available due to ethical, privacy, and third party restrictions. Data are however available from the authors upon reasonable request and with permission of the TAKECARE consortium (the Province of Groningen, the University Medical Center Groningen, the University of Groningen, the Menzis health insurance company and care organizations Elker and Accare) and the Netherlands Organisation of Applied Scientific Research (TNO).

## Abbreviations

BB: Beta binomial
BCPE: Box-Cox power exponential
BIC: Bayes Information Criterion
GAMLSS: Generalized additive models for location, scale, and shape
SDQ: Strengths and Difficulties Questionnaire
UK: United Kingdom
