# Supplementary material for: Normative data for the self-reported and parent-reported Strengths and Difficulties Questionnaire (SDQ) for ages 12–17
Source: Child Adolesc Psychiatry Ment Health. 2022 Jan 18;16:5. doi: 10.1186/s13034-021-00437-8 (PMC8764849; doi:10.1186/s13034-021-00437-8)
Supplement: Supplementary file 1 — Additional file 1: (‘Additional file 1.pdf’) contains information on the weighting applied to correct over overrepresentation of females and adolescents with a Dutch background in the samples used to establish norms for the Dutch self-reported and parent-reported SDQ versions. [file 13034_2021_437_MOESM1_ESM.pdf]

This file is supplemental to manuscript:

Normative data for the self-reported and parent-reported Strengths and Difficulties Questionnaire (SDQ) for ages 12-17

Contents

The file contains information on the weighting applied to correct over overrepresentation of females and adolescents with a Dutch background in the samples used to establish norms for the Dutch self-reported and parent-reported SDQ versions. The method for calculating weights was the same for both SDQ versions. For the sake of conciseness we only thoroughly explain how the weights for the self-reported version were calculated.

Weighting

The sample characteristics and, for comparison, the population characteristics are presented in Table 1 of the manuscript. The population characteristics reflect the desired representation per characteristic. The table shows that females and adolescents with a Dutch background were overrepresented in the samples used for establishing norms for the self-reported and the parent-reported SDQ versions. These overrepresentations were corrected for by applying the weights presented in Table S1 in this document. In this document we explain how we calculated the weights for the self-reported SDQ version.

Table S1 Per SDQ version and type of norms (gender-specific or joint): weights used to correct for oversampling of females and adolescents with a Dutch background

		SDQ informant version			
		Self-report		Parent-report	
Ethnic background	Gender	Gender-specific norms	Joint norms	Gender-specific norms	Joint norms
Dutch	Male	0.45	0.45	0.37	0.37
	Female	0.45	0.40	0.37	0.33
Other than Dutch	Male	1	1	1	1
	Female	1	0.90	1	0.89

SDQ = Strengths and Difficulties Questionnaire

Weights for the calculation of gender-specific norms

For calculating the gender-specific norms, we corrected for the overrepresentation of adolescents with a Dutch background. Note that correcting for the overrepresentation of females was not necessary, because these gender-specific norms were calculated for male and female adolescents separately.

Table 1 shows that the desired percentage of adolescents with a Dutch ethnic background is 78.6%, and 21.4% with an other than Dutch ethnic background. The sample contained 89.1% (n = 885) adolescents with a Dutch ethnic background, and 10.9% (n =108) with an other than Dutch ethnic background (n =108). To correct for the overrepresentation of the Dutch background adolescents, the scores of the Dutch background adolescents were downweighted, where the weight was computed as (78.6*10.9)/(21.4*89.1) = .45, and the scores of adolescents with other than Dutch background were weighted with 1. This ensures that the relative weight of the Dutch background adolescents in the norming is 78.6%, as desired.

Weighting for the calculation of joint norms

For the calculation of the joint norms, we correct both for the overrepresentation of adolescents with a Dutch background and for the overrepresentation of females.

Table 1 shows that the desired percentage of females is 50.5%. The sample contained 53.1% females (n = 527) and 46.9% males (n = 466).

To correct for the overrepresentation of the females, the scores of the females were downweighted, where the weights for the females was computed as (50.5*46.9)/(49.5*53.1) = .90, and the scores of males were weighted with 1. We combined these gender weights with the ethnic background weights (0.45 for ‘Dutch’ and 1 for ‘other’) by multiplying the weights belonging to the specific combination, thereby assuming that the gender and ethnic background underrepresentations are independent. This revealed the following weights: Females with a Dutch ethnic background .90 * .45 = .40; Males with Dutch ethnic background: 1 * .45 = .45; Females with an other than Dutch ethnic background .90 * 1 = .90; Males with an other than Dutch ethnic background 1 * 1 = 1. To assess the reasonableness of the independency assumption, we verified that the distributions using these weighted percentages for gender and ethnic background are similar to the target figures.
