# Supplementary material for: Normative data for the self-reported and parent-reported Strengths and Difficulties Questionnaire (SDQ) for ages 12–17
Source: Child Adolesc Psychiatry Ment Health. 2022 Jan 18;16:5. doi: 10.1186/s13034-021-00437-8 (PMC8764849; doi:10.1186/s13034-021-00437-8)
Supplement: Supplementary file 2 — Additional file 2: (‘Additional file 2.pdf’) contains the complete norms (gender-specific and joint) per year of age for the self-reported and parent-reported SDQ versions. [file 13034_2021_437_MOESM2_ESM.pdf]

This file is supplemental to manuscript:

Normative data for the self-reported and parent-reported Strengths and Difficulties Questionnaire (SDQ) for ages 12-17

**Contents**

This file contains the complete norms for both SDQ versions.

*Table S1 Norms (percentiles) for the self-reported SDQ version for female adolescents aged 12 to 17*

| | | **Scale score** | | | | | | | | | | |
|---|---|---|---|---|---|---|---|---|---|---|---|---|
| SDQ Scale | Age | **0** | **1** | **2** | **3** | **4** | **5** | **6** | **7** | **8** | **9** | **10** |
| Emotional | 12 | 25.2 | 44.5 | 59.7 | 71.7 | 81.0 | 88.0 | 93.0 | 96.4 | 98.5 | 99.6 | 100 |
| | 13 | 21.6 | 40.6 | 56.6 | 69.5 | 79.7 | 87.3 | 92.8 | 96.4 | 98.6 | 99.7 | 100 |
| | 14 | 18.2 | 36.7 | 53.3 | 67.2 | 78.2 | 86.6 | 92.5 | 96.4 | 98.6 | 99.7 | 100 |
| | 15 | 15.1 | 32.8 | 49.8 | 64.5 | 76.5 | 85.7 | 92.1 | 96.3 | 98.6 | 99.7 | 100 |
| | 16 | 12.4 | 29.0 | 46.1 | 61.7 | 74.6 | 84.6 | 91.7 | 96.2 | 98.6 | 99.7 | 100 |
| | 17 | 10.0 | 25.3 | 42.3 | 58.6 | 72.5 | 83.4 | 91.1 | 96.0 | 98.6 | 99.7 | 100 |
| Conduct | 12 | 30.4 | 63.0 | 84.0 | 94.3 | 98.3 | 99.6 | 99.9 | 100 | 100 | 100 | 100 |
| | 13 | 31.3 | 64.4 | 85.1 | 94.9 | 98.5 | 99.7 | 99.9 | 100 | 100 | 100 | 100 |
| | 14 | 32.3 | 65.8 | 86.2 | 95.4 | 98.7 | 99.7 | 99.9 | 100 | 100 | 100 | 100 |
| | 15 | 33.4 | 67.1 | 87.1 | 95.9 | 98.9 | 99.8 | 100 | 100 | 100 | 100 | 100 |
| | 16 | 34.4 | 68.5 | 88.1 | 96.3 | 99.1 | 99.8 | 100 | 100 | 100 | 100 | 100 |
| | 17 | 35.5 | 69.8 | 89.0 | 96.7 | 99.2 | 99.8 | 100 | 100 | 100 | 100 | 100 |
| Hyperactivity | 12 | 9.5 | 23.7 | 39.4 | 54.8 | 68.5 | 79.8 | 88.3 | 94.2 | 97.8 | 99.5 | 100 |
| | 13 | 9.9 | 24.1 | 39.6 | 54.7 | 68.2 | 79.4 | 87.9 | 93.9 | 97.6 | 99.4 | 100 |
| | 14 | 10.3 | 24.5 | 39.8 | 54.7 | 67.9 | 78.9 | 87.5 | 93.6 | 97.4 | 99.3 | 100 |
| | 15 | 10.7 | 24.9 | 40.1 | 54.6 | 67.6 | 78.5 | 87.0 | 93.2 | 97.2 | 99.3 | 100 |
| | 16 | 11.1 | 25.4 | 40.3 | 54.6 | 67.3 | 78.1 | 86.6 | 92.8 | 96.9 | 99.2 | 100 |
| | 17 | 11.5 | 25.8 | 40.6 | 54.5 | 67.1 | 77.7 | 86.1 | 92.5 | 96.7 | 99.1 | 100 |
| Social | 12 | 44.1 | 67.6 | 81.6 | 90.0 | 94.9 | 97.6 | 99.0 | 99.6 | 99.9 | 100 | 100 |
| | 13 | 41.9 | 66.5 | 81.4 | 90.2 | 95.2 | 97.8 | 99.1 | 99.7 | 99.9 | 100 | 100 |
| | 14 | 39.7 | 65.3 | 81.1 | 90.3 | 95.4 | 98.0 | 99.3 | 99.8 | 99.9 | 100 | 100 |
| | 15 | 37.6 | 64.1 | 80.8 | 90.5 | 95.7 | 98.2 | 99.4 | 99.8 | 100 | 100 | 100 |
| | 16 | 35.5 | 62.9 | 80.5 | 90.6 | 95.9 | 98.4 | 99.5 | 99.9 | 100 | 100 | 100 |
| | 17 | 33.6 | 61.7 | 80.1 | 90.7 | 96.1 | 98.5 | 99.5 | 99.9 | 100 | 100 | 100 |
| Prosocial | 12 | 100 | 100 | 99.9 | 99.7 | 98.9 | 96.4 | 90.1 | 77.0 | 54.3 | 24.4 | 0.0 |
| | 13 | 100 | 100 | 99.9 | 99.7 | 98.8 | 96.1 | 89.7 | 76.5 | 54.2 | 24.7 | 0.0 |
| | 14 | 100 | 100 | 99.9 | 99.6 | 98.6 | 95.8 | 89.2 | 76.1 | 54.2 | 25.1 | 0.0 |
| | 15 | 100 | 100 | 99.9 | 99.6 | 98.4 | 95.5 | 88.7 | 75.7 | 54.1 | 25.4 | 0.0 |
| | 16 | 100 | 100 | 99.9 | 99.5 | 98.2 | 95.1 | 88.3 | 75.3 | 54.1 | 25.9 | 0.0 |
| | 17 | 100 | 100 | 99.8 | 99.4 | 98.0 | 94.7 | 87.7 | 74.8 | 54.1 | 26.3 | 0.0 |

SDQ = Strengths and Difficulties Questionnaire.
Cells containing percentiles ≥ 90 are shaded dark gray and cells containing percentiles from 80 up to 90 light gray, with the corresponding scale scores classified as falling in the 'abnormal' and 'borderline' range, respectively. The remaining scale scores are classified as 'normal'.

*Table S1 (continued) Norms (percentiles) for the self-reported SDQ version for female adolescents aged 12 to 17*

| | | Scale score | | | | | | | | | | | | | | | | | | | | |
|---|---|---|---|---|---|---|---|---|---|---|---|---|---|---|---|---|---|---|---|---|---|---|
| **SDQ Scale** | **Age** | **0** | **1** | **2** | **3** | **4** | **5** | **6** | **7** | **8** | **9** | **10** | **11** | **12** | **13** | **14** | **15** | **16** | **17** | **18** | **19** | **20** |
| Externalizing | 12 | 5.4 | 15.0 | 27.1 | 40.0 | 52.5 | 63.8 | 73.4 | 81.2 | 87.2 | 91.7 | 94.8 | 97.0 | 98.3 | 99.1 | 99.6 | 99.8 | 99.9 | 100 | 100 | 100 | 100 |
| | 13 | 5.6 | 15.5 | 27.7 | 40.6 | 53.1 | 64.2 | 73.7 | 81.4 | 87.3 | 91.7 | 94.8 | 96.9 | 98.3 | 99.1 | 99.6 | 99.8 | 99.9 | 100 | 100 | 100 | 100 |
| | 14 | 5.9 | 16.0 | 28.3 | 41.2 | 53.6 | 64.6 | 74.0 | 81.5 | 87.4 | 91.7 | 94.8 | 96.9 | 98.3 | 99.1 | 99.6 | 99.8 | 99.9 | 100 | 100 | 100 | 100 |
| | 15 | 6.1 | 16.5 | 28.9 | 41.8 | 54.1 | 65.0 | 74.2 | 81.7 | 87.5 | 91.8 | 94.8 | 96.9 | 98.3 | 99.1 | 99.6 | 99.8 | 99.9 | 100 | 100 | 100 | 100 |
| | 16 | 6.4 | 17.0 | 29.5 | 42.4 | 54.6 | 65.4 | 74.5 | 81.9 | 87.6 | 91.8 | 94.8 | 96.9 | 98.3 | 99.1 | 99.6 | 99.8 | 99.9 | 100 | 100 | 100 | 100 |
| | 17 | 6.7 | 17.5 | 30.1 | 43.0 | 55.1 | 65.8 | 74.8 | 82.0 | 87.6 | 91.8 | 94.8 | 96.9 | 98.2 | 99.1 | 99.5 | 99.8 | 99.9 | 100 | 100 | 100 | 100 |
| Internalizing | 12 | 17.0 | 32.5 | 46.0 | 57.4 | 66.9 | 74.7 | 81.1 | 86.1 | 90.0 | 93.0 | 95.3 | 96.9 | 98.1 | 98.8 | 99.4 | 99.7 | 99.8 | 99.9 | 100 | 100 | 100 |
| | 13 | 14.0 | 28.7 | 42.4 | 54.4 | 64.7 | 73.2 | 80.1 | 85.6 | 89.8 | 93.0 | 95.4 | 97.1 | 98.2 | 99.0 | 99.4 | 99.7 | 99.9 | 100 | 100 | 100 | 100 |
| | 14 | 11.3 | 25.0 | 38.7 | 51.3 | 62.3 | 71.5 | 79.0 | 85.0 | 89.6 | 93.0 | 95.5 | 97.2 | 98.3 | 99.1 | 99.5 | 99.8 | 99.9 | 100 | 100 | 100 | 100 |
| | 15 | 9.0 | 21.5 | 35.0 | 48.0 | 59.7 | 69.6 | 77.8 | 84.3 | 89.2 | 92.9 | 95.5 | 97.3 | 98.4 | 99.2 | 99.6 | 99.8 | 99.9 | 100 | 100 | 100 | 100 |
| | 16 | 7.0 | 18.2 | 31.3 | 44.5 | 56.8 | 67.5 | 76.4 | 83.5 | 88.8 | 92.8 | 95.5 | 97.4 | 98.5 | 99.2 | 99.6 | 99.8 | 99.9 | 100 | 100 | 100 | 100 |
| | 17 | 5.4 | 15.2 | 27.6 | 40.9 | 53.7 | 65.2 | 74.8 | 82.5 | 88.3 | 92.5 | 95.5 | 97.4 | 98.6 | 99.3 | 99.7 | 99.9 | 100 | 100 | 100 | 100 | 100 |
| | | **0** | **1** | **2** | **3** | **4** | **5** | **6** | **7** | **8** | **9** | **10** | **11** | **12** | **13** | **14** | **15** | **16** | **17** | **18** | **19** | **20** |
| Total | 12 | 0.0 | 2.8 | 6.6 | 11.6 | 18.0 | 25.4 | 33.8 | 42.7 | 51.7 | 60.0 | 67.1 | 73.2 | 78.2 | 82.4 | 85.8 | 88.6 | 90.8 | 92.6 | 94.1 | 95.3 | 96.2 |
| | 13 | 0.0 | 2.8 | 6.5 | 11.5 | 17.8 | 25.1 | 33.4 | 42.2 | 51.2 | 59.5 | 66.7 | 72.8 | 77.8 | 82.0 | 85.5 | 88.3 | 90.6 | 92.4 | 93.9 | 95.1 | 96.1 |
| | 14 | 0.0 | 2.7 | 6.4 | 11.4 | 17.6 | 24.9 | 33.0 | 41.8 | 50.7 | 59.0 | 66.2 | 72.3 | 77.4 | 81.7 | 85.2 | 88.0 | 90.3 | 92.2 | 93.8 | 95.0 | 96.0 |
| | 15 | 0.0 | 2.7 | 6.4 | 11.3 | 17.4 | 24.6 | 32.7 | 41.3 | 50.2 | 58.5 | 65.7 | 71.9 | 77.0 | 81.3 | 84.9 | 87.8 | 90.1 | 92.0 | 93.6 | 94.8 | 95.8 |
| | 16 | 0.0 | 2.7 | 6.3 | 11.2 | 17.2 | 24.3 | 32.3 | 40.9 | 49.7 | 58.0 | 65.2 | 71.4 | 76.6 | 81.0 | 84.5 | 87.5 | 89.9 | 91.8 | 93.4 | 94.7 | 95.7 |
| | 17 | 0.0 | 2.7 | 6.3 | 11.0 | 17.0 | 24.0 | 32.0 | 40.5 | 49.2 | 57.5 | 64.8 | 71.0 | 76.2 | 80.6 | 84.2 | 87.2 | 89.6 | 91.6 | 93.2 | 94.5 | 95.6 |
| | | **21** | **22** | **23** | **24** | **25** | **26** | **27** | **28** | **29** | **30** | **31** | **32** | **33** | **34** | **35** | **36** | **37** | **38** | **39** | **40** | |
| | 12 | 97.0 | 97.6 | 98.1 | 98.5 | 98.8 | 99.0 | 99.2 | 99.4 | 99.5 | 99.6 | 99.7 | 99.7 | 99.8 | 99.8 | 99.9 | 99.9 | 99.9 | 99.9 | 99.9 | 100 | |
| | 13 | 96.9 | 97.5 | 98.0 | 98.4 | 98.7 | 99.0 | 99.2 | 99.3 | 99.5 | 99.6 | 99.7 | 99.7 | 99.8 | 99.8 | 99.9 | 99.9 | 99.9 | 99.9 | 99.9 | 100 | |
| | 14 | 96.8 | 97.4 | 97.9 | 98.3 | 98.7 | 98.9 | 99.1 | 99.3 | 99.4 | 99.6 | 99.6 | 99.7 | 99.8 | 99.8 | 99.9 | 99.9 | 99.9 | 99.9 | 99.9 | 100 | |
| | 15 | 96.7 | 97.3 | 97.8 | 98.3 | 98.6 | 98.9 | 99.1 | 99.3 | 99.4 | 99.5 | 99.6 | 99.7 | 99.8 | 99.8 | 99.8 | 99.9 | 99.9 | 99.9 | 99.9 | 99.9 | |
| | 16 | 96.5 | 97.2 | 97.8 | 98.2 | 98.6 | 98.8 | 99.1 | 99.2 | 99.4 | 99.5 | 99.6 | 99.7 | 99.7 | 99.8 | 99.8 | 99.9 | 99.9 | 99.9 | 99.9 | 99.9 | |
| | 17 | 96.4 | 97.1 | 97.7 | 98.1 | 98.5 | 98.8 | 99.0 | 99.2 | 99.4 | 99.5 | 99.6 | 99.7 | 99.7 | 99.8 | 99.8 | 99.9 | 99.9 | 99.9 | 99.9 | 99.9 | |

SDQ = Strengths and Difficulties Questionnaire.
Cells containing percentiles ≥ 90 are shaded dark gray and cells containing percentiles from 80 up to 90 light gray, with the corresponding scale scores classified as falling in the 'abnormal' and 'borderline' range, respectively. The remaining scale scores are classified as 'normal'.

*Table S2 Norms (percentiles) for the self-reported SDQ version for male adolescents aged 12 to 17*

| | | **Scale score** | | | | | | | | | | |
|---|---|---|---|---|---|---|---|---|---|---|---|---|
| SDQ Scale | Age | **0** | **1** | **2** | **3** | **4** | **5** | **6** | **7** | **8** | **9** | **10** |
| Emotional | 12 | 37.5 | 61.0 | 76.3 | 86.3 | 92.5 | 96.2 | 98.3 | 99.4 | 99.8 | 100 | 100 |
| | 13 | 33.6 | 57.7 | 74.3 | 85.3 | 92.1 | 96.1 | 98.3 | 99.4 | 99.8 | 100 | 100 |
| | 14 | 29.8 | 54.3 | 72.1 | 84.1 | 91.6 | 96.0 | 98.3 | 99.4 | 99.8 | 100 | 100 |
| | 15 | 26.1 | 50.8 | 69.7 | 82.8 | 91.0 | 95.8 | 98.3 | 99.4 | 99.9 | 100 | 100 |
| | 16 | 22.7 | 47.1 | 67.0 | 81.2 | 90.3 | 95.5 | 98.2 | 99.4 | 99.9 | 100 | 100 |
| | 17 | 19.5 | 43.3 | 64.1 | 79.5 | 89.5 | 95.2 | 98.1 | 99.4 | 99.9 | 100 | 100 |
| Conduct | 12 | 24.8 | 53.0 | 74.5 | 87.9 | 95.0 | 98.2 | 99.5 | 99.9 | 100 | 100 | 100 |
| | 13 | 25.5 | 54.3 | 75.9 | 88.9 | 95.6 | 98.5 | 99.6 | 99.9 | 100 | 100 | 100 |
| | 14 | 26.3 | 55.7 | 77.2 | 89.9 | 96.1 | 98.7 | 99.7 | 99.9 | 100 | 100 | 100 |
| | 15 | 27.1 | 57.0 | 78.5 | 90.8 | 96.6 | 98.9 | 99.7 | 99.9 | 100 | 100 | 100 |
| | 16 | 27.9 | 58.4 | 79.8 | 91.6 | 97.0 | 99.1 | 99.8 | 100 | 100 | 100 | 100 |
| | 17 | 28.7 | 59.7 | 81.0 | 92.4 | 97.4 | 99.3 | 99.8 | 100 | 100 | 100 | 100 |
| Hyperactivity | 12 | 6.6 | 18.5 | 33.4 | 49.1 | 64.0 | 76.7 | 86.6 | 93.4 | 97.5 | 99.4 | 100 |
| | 13 | 6.9 | 18.8 | 33.6 | 49.1 | 63.7 | 76.3 | 86.1 | 93.1 | 97.3 | 99.4 | 100 |
| | 14 | 7.2 | 19.2 | 33.9 | 49.1 | 63.5 | 75.9 | 85.7 | 92.7 | 97.1 | 99.3 | 100 |
| | 15 | 7.5 | 19.6 | 34.1 | 49.1 | 63.2 | 75.5 | 85.2 | 92.3 | 96.9 | 99.2 | 100 |
| | 16 | 7.8 | 20.0 | 34.4 | 49.1 | 63.0 | 75.0 | 84.8 | 92.0 | 96.6 | 99.1 | 100 |
| | 17 | 8.1 | 20.5 | 34.7 | 49.1 | 62.7 | 74.6 | 84.3 | 91.6 | 96.4 | 99.0 | 100 |
| Social | 12 | 39.8 | 63.1 | 77.9 | 87.3 | 93.1 | 96.6 | 98.5 | 99.4 | 99.8 | 100 | 100 |
| | 13 | 37.5 | 61.7 | 77.4 | 87.4 | 93.4 | 96.8 | 98.7 | 99.5 | 99.9 | 100 | 100 |
| | 14 | 35.2 | 60.3 | 77.0 | 87.5 | 93.7 | 97.1 | 98.8 | 99.6 | 99.9 | 100 | 100 |
| | 15 | 33.1 | 58.9 | 76.5 | 87.5 | 93.9 | 97.3 | 99.0 | 99.7 | 99.9 | 100 | 100 |
| | 16 | 31.0 | 57.5 | 76.0 | 87.6 | 94.1 | 97.5 | 99.1 | 99.7 | 99.9 | 100 | 100 |
| | 17 | 29.0 | 56.1 | 75.5 | 87.6 | 94.4 | 97.7 | 99.2 | 99.8 | 100 | 100 | 100 |
| prosocial | 12 | 100 | 99.9 | 99.6 | 98.4 | 95.4 | 88.9 | 77.2 | 59.2 | 36.2 | 13.7 | 0.0 |
| | 13 | 100 | 99.9 | 99.5 | 98.2 | 95.0 | 88.4 | 76.6 | 58.9 | 36.4 | 14.1 | 0.0 |
| | 14 | 100 | 99.9 | 99.4 | 98.0 | 94.6 | 87.8 | 76.1 | 58.6 | 36.6 | 14.5 | 0.0 |
| | 15 | 100 | 99.8 | 99.3 | 97.7 | 94.1 | 87.2 | 75.5 | 58.4 | 36.9 | 14.9 | 0.0 |
| | 16 | 100 | 99.8 | 99.2 | 97.4 | 93.7 | 86.6 | 75.0 | 58.2 | 37.2 | 15.4 | 0.0 |
| | 17 | 100 | 99.7 | 99.0 | 97.1 | 93.1 | 85.9 | 74.4 | 58.0 | 37.5 | 16.0 | 0.0 |

SDQ = Strengths and Difficulties Questionnaire.
Cells containing percentiles ≥ 90 are shaded dark gray and cells containing percentiles from 80 up to 90 light gray, with the corresponding scale scores classified as falling in the 'abnormal' and 'borderline' range, respectively. The remaining scale scores are classified as 'normal'.

*Table S2 (continued) Norms (percentiles) for the self-reported SDQ version for male adolescents aged 12 to 17*

| | | Scale score | | | | | | | | | | | | | | | | | | | | |
|---|---|---|---|---|---|---|---|---|---|---|---|---|---|---|---|---|---|---|---|---|---|---|
| **SDQ Scale** | **Age** | **0** | **1** | **2** | **3** | **4** | **5** | **6** | **7** | **8** | **9** | **10** | **11** | **12** | **13** | **14** | **15** | **16** | **17** | **18** | **19** | **20** |
| Externalizing | 12 | 3.1 | 10.0 | 19.8 | 31.5 | 43.7 | 55.6 | 66.3 | 75.5 | 82.9 | 88.6 | 92.7 | 95.6 | 97.5 | 98.7 | 99.4 | 99.7 | 99.9 | 100 | 100 | 100 | 100 |
| | 13 | 3.3 | 10.4 | 20.4 | 32.1 | 44.3 | 56.1 | 66.7 | 75.7 | 83.0 | 88.7 | 92.8 | 95.6 | 97.5 | 98.7 | 99.4 | 99.7 | 99.9 | 100 | 100 | 100 | 100 |
| | 14 | 3.5 | 10.8 | 20.9 | 32.7 | 44.9 | 56.6 | 67.1 | 76.0 | 83.2 | 88.7 | 92.8 | 95.7 | 97.5 | 98.7 | 99.4 | 99.7 | 99.9 | 100 | 100 | 100 | 100 |
| | 15 | 3.7 | 11.2 | 21.5 | 33.3 | 45.5 | 57.1 | 67.4 | 76.2 | 83.3 | 88.8 | 92.8 | 95.7 | 97.5 | 98.7 | 99.4 | 99.7 | 99.9 | 100 | 100 | 100 | 100 |
| | 16 | 3.9 | 11.6 | 22.0 | 33.9 | 46.0 | 57.6 | 67.8 | 76.5 | 83.5 | 88.9 | 92.9 | 95.7 | 97.5 | 98.7 | 99.4 | 99.7 | 99.9 | 100 | 100 | 100 | 100 |
| | 17 | 4.1 | 12.0 | 22.6 | 34.5 | 46.6 | 58.0 | 68.2 | 76.7 | 83.6 | 89.0 | 92.9 | 95.7 | 97.5 | 98.7 | 99.3 | 99.7 | 99.9 | 100 | 100 | 100 | 100 |
| Internalizing | 12 | 22.5 | 40.6 | 55.1 | 66.5 | 75.5 | 82.3 | 87.5 | 91.4 | 94.2 | 96.2 | 97.6 | 98.5 | 99.2 | 99.5 | 99.8 | 99.9 | 100 | 100 | 100 | 100 | 100 |
| | 13 | 19.1 | 36.9 | 51.9 | 64.2 | 73.9 | 81.4 | 87.1 | 91.2 | 94.2 | 96.3 | 97.7 | 98.7 | 99.3 | 99.6 | 99.8 | 99.9 | 100 | 100 | 100 | 100 | 100 |
| | 14 | 16.0 | 33.1 | 48.6 | 61.7 | 72.2 | 80.4 | 86.5 | 91.0 | 94.2 | 96.4 | 97.9 | 98.8 | 99.3 | 99.7 | 99.8 | 99.9 | 100 | 100 | 100 | 100 | 100 |
| | 15 | 13.2 | 29.4 | 45.2 | 59.0 | 70.3 | 79.2 | 85.9 | 90.8 | 94.2 | 96.5 | 98.0 | 98.9 | 99.4 | 99.7 | 99.9 | 100 | 100 | 100 | 100 | 100 | 100 |
| | 16 | 10.7 | 25.8 | 41.6 | 56.0 | 68.2 | 77.8 | 85.1 | 90.4 | 94.1 | 96.5 | 98.0 | 98.9 | 99.5 | 99.8 | 99.9 | 100 | 100 | 100 | 100 | 100 | 100 |
| | 17 | 8.6 | 22.4 | 38.0 | 52.9 | 65.8 | 76.3 | 84.2 | 90.0 | 93.9 | 96.5 | 98.1 | 99.0 | 99.5 | 99.8 | 99.9 | 100 | 100 | 100 | 100 | 100 | 100 |
| | | **0** | **1** | **2** | **3** | **4** | **5** | **6** | **7** | **8** | **9** | **10** | **11** | **12** | **13** | **14** | **15** | **16** | **17** | **18** | **19** | **20** |
| Total | 12 | 0.0 | 2.4 | 6.2 | 11.6 | 18.3 | 26.0 | 34.5 | 43.1 | 51.6 | 59.5 | 66.7 | 73.0 | 78.3 | 82.8 | 86.5 | 89.5 | 92.0 | 93.9 | 95.4 | 96.5 | 97.4 |
| | 13 | 0.0 | 2.4 | 6.2 | 11.5 | 18.1 | 25.8 | 34.1 | 42.7 | 51.1 | 59.1 | 66.2 | 72.5 | 77.9 | 82.4 | 86.2 | 89.3 | 91.7 | 93.7 | 95.2 | 96.4 | 97.3 |
| | 14 | 0.0 | 2.4 | 6.1 | 11.3 | 17.9 | 25.5 | 33.7 | 42.2 | 50.7 | 58.6 | 65.7 | 72.1 | 77.5 | 82.1 | 85.9 | 89.0 | 91.5 | 93.4 | 95.0 | 96.2 | 97.2 |
| | 15 | 0.0 | 2.4 | 6.1 | 11.2 | 17.7 | 25.2 | 33.4 | 41.8 | 50.2 | 58.1 | 65.3 | 71.6 | 77.1 | 81.7 | 85.5 | 88.7 | 91.2 | 93.2 | 94.8 | 96.1 | 97.0 |
| | 16 | 0.0 | 2.4 | 6.0 | 11.1 | 17.5 | 24.9 | 33.0 | 41.4 | 49.7 | 57.6 | 64.8 | 71.1 | 76.6 | 81.3 | 85.2 | 88.4 | 90.9 | 93.0 | 94.6 | 95.9 | 96.9 |
| | 17 | 0.0 | 2.3 | 5.9 | 11.0 | 17.3 | 24.6 | 32.7 | 41.0 | 49.3 | 57.1 | 64.3 | 70.7 | 76.2 | 80.9 | 84.8 | 88.1 | 90.7 | 92.8 | 94.5 | 95.8 | 96.8 |
| | | **21** | **22** | **23** | **24** | **25** | **26** | **27** | **28** | **29** | **30** | **31** | **32** | **33** | **34** | **35** | **36** | **37** | **38** | **39** | **40** | |
| | 12 | 98.1 | 98.6 | 99.0 | 99.2 | 99.4 | 99.6 | 99.7 | 99.8 | 99.9 | 99.9 | 99.9 | 99.9 | 100 | 100 | 100 | 100 | 100 | 100 | 100 | 100 | |
| | 13 | 98.0 | 98.5 | 98.9 | 99.2 | 99.4 | 99.6 | 99.7 | 99.8 | 99.8 | 99.9 | 99.9 | 99.9 | 100 | 100 | 100 | 100 | 100 | 100 | 100 | 100 | |
| | 14 | 97.9 | 98.4 | 98.8 | 99.2 | 99.4 | 99.6 | 99.7 | 99.8 | 99.8 | 99.9 | 99.9 | 99.9 | 100 | 100 | 100 | 100 | 100 | 100 | 100 | 100 | |
| | 15 | 97.8 | 98.4 | 98.8 | 99.1 | 99.3 | 99.5 | 99.7 | 99.8 | 99.8 | 99.9 | 99.9 | 99.9 | 100 | 100 | 100 | 100 | 100 | 100 | 100 | 100 | |
| | 16 | 97.7 | 98.3 | 98.7 | 99.1 | 99.3 | 99.5 | 99.6 | 99.7 | 99.8 | 99.9 | 99.9 | 99.9 | 100 | 100 | 100 | 100 | 100 | 100 | 100 | 100 | |
| | 17 | 97.6 | 98.2 | 98.7 | 99.0 | 99.3 | 99.5 | 99.6 | 99.7 | 99.8 | 99.9 | 99.9 | 99.9 | 99.9 | 100 | 100 | 100 | 100 | 100 | 100 | 100 | |

SDQ = Strengths and Difficulties Questionnaire.
Cells containing percentiles ≥ 90 are shaded dark gray and cells containing percentiles from 80 up to 90 light gray, with the corresponding scale scores classified as falling in the 'abnormal' and 'borderline' range, respectively. The remaining scale scores are classified as 'normal'.

*Table S3 Norms (percentiles) for the self-reported SDQ version for adolescents aged 12 to 17, without distinguishing between genders*

| | | Scale score | | | | | | | | | | |
|---|---|---|---|---|---|---|---|---|---|---|---|---|
| SDQ Scale | Age | **0** | **1** | **2** | **3** | **4** | **5** | **6** | **7** | **8** | **9** | **10** |
| Emotional | 12 | 31.3 | 52.9 | 68.6 | 79.8 | 87.7 | 93.0 | 96.4 | 98.4 | 99.4 | 99.9 | 100 |
| | 13 | 27.7 | 49.3 | 65.7 | 77.8 | 86.4 | 92.3 | 96.1 | 98.3 | 99.4 | 99.9 | 100 |
| | 14 | 24.3 | 45.5 | 62.5 | 75.6 | 85.0 | 91.5 | 95.7 | 98.1 | 99.4 | 99.9 | 100 |
| | 15 | 21.0 | 41.6 | 59.2 | 73.1 | 83.4 | 90.6 | 95.2 | 97.9 | 99.3 | 99.9 | 100 |
| | 16 | 18.0 | 37.7 | 55.7 | 70.3 | 81.5 | 89.5 | 94.6 | 97.7 | 99.2 | 99.8 | 100 |
| | 17 | 15.2 | 33.9 | 51.9 | 67.3 | 79.4 | 88.2 | 94.0 | 97.4 | 99.1 | 99.8 | 100 |
| Conduct | 12 | 27.3 | 57.3 | 78.7 | 90.8 | 96.6 | 98.9 | 99.7 | 99.9 | 100 | 100 | 100 |
| | 13 | 28.3 | 58.9 | 80.1 | 91.8 | 97.1 | 99.1 | 99.8 | 100 | 100 | 100 | 100 |
| | 14 | 29.3 | 60.4 | 81.5 | 92.6 | 97.5 | 99.3 | 99.8 | 100 | 100 | 100 | 100 |
| | 15 | 30.4 | 62.0 | 82.8 | 93.4 | 97.8 | 99.4 | 99.9 | 100 | 100 | 100 | 100 |
| | 16 | 31.5 | 63.5 | 84.0 | 94.1 | 98.2 | 99.5 | 99.9 | 100 | 100 | 100 | 100 |
| | 17 | 32.6 | 65.1 | 85.2 | 94.7 | 98.4 | 99.6 | 99.9 | 100 | 100 | 100 | 100 |
| Hyperactivity | 12 | 8.0 | 20.9 | 36.2 | 51.7 | 66.0 | 78.0 | 87.2 | 93.7 | 97.6 | 99.4 | 100 |
| | 13 | 8.3 | 21.4 | 36.5 | 51.8 | 65.8 | 77.7 | 86.9 | 93.4 | 97.4 | 99.4 | 100 |
| | 14 | 8.7 | 21.8 | 36.8 | 51.9 | 65.7 | 77.4 | 86.5 | 93.1 | 97.2 | 99.3 | 100 |
| | 15 | 9.0 | 22.3 | 37.2 | 52.0 | 65.5 | 77.0 | 86.2 | 92.8 | 97.0 | 99.2 | 100 |
| | 16 | 9.4 | 22.8 | 37.5 | 52.1 | 65.4 | 76.7 | 85.8 | 92.5 | 96.8 | 99.2 | 100 |
| | 17 | 9.8 | 23.2 | 37.8 | 52.1 | 65.2 | 76.4 | 85.4 | 92.1 | 96.6 | 99.1 | 100 |
| Social | 12 | 41.6 | 65.0 | 79.4 | 88.4 | 93.8 | 97.0 | 98.7 | 99.5 | 99.9 | 100 | 100 |
| | 13 | 39.5 | 63.8 | 79.2 | 88.6 | 94.2 | 97.3 | 98.9 | 99.6 | 99.9 | 100 | 100 |
| | 14 | 37.4 | 62.7 | 78.9 | 88.8 | 94.5 | 97.6 | 99.0 | 99.7 | 99.9 | 100 | 100 |
| | 15 | 35.4 | 61.6 | 78.7 | 89.0 | 94.8 | 97.8 | 99.2 | 99.8 | 99.9 | 100 | 100 |
| | 16 | 33.5 | 60.5 | 78.4 | 89.2 | 95.1 | 98.0 | 99.3 | 99.8 | 100 | 100 | 100 |
| | 17 | 31.7 | 59.3 | 78.2 | 89.4 | 95.4 | 98.2 | 99.4 | 99.8 | 100 | 100 | 100 |
| prosocial | 12 | 100 | 100 | 99.8 | 99.1 | 97.1 | 92.5 | 83.1 | 67.1 | 44.1 | 18.4 | 0.0 |
| | 13 | 100 | 100 | 99.7 | 99.0 | 96.9 | 92.1 | 82.8 | 67.0 | 44.6 | 19.1 | 0.0 |
| | 14 | 100 | 99.9 | 99.7 | 98.8 | 96.6 | 91.7 | 82.4 | 67.0 | 45.1 | 19.8 | 0.0 |
| | 15 | 100 | 99.9 | 99.6 | 98.7 | 96.3 | 91.3 | 82.1 | 67.0 | 45.6 | 20.6 | 0.0 |
| | 16 | 100 | 99.9 | 99.5 | 98.5 | 96.0 | 90.9 | 81.7 | 67.0 | 46.2 | 21.4 | 0.0 |
| | 17 | 100 | 99.9 | 99.5 | 98.3 | 95.7 | 90.5 | 81.4 | 67.0 | 46.8 | 22.3 | 0.0 |

SDQ = Strengths and Difficulties Questionnaire.
Cells containing percentiles ≥ 90 are shaded dark gray and cells containing percentiles from 80 up to 90 light gray, with the corresponding scale scores classified as falling in the ‘abnormal’ and ‘borderline’ range, respectively. The remaining scale scores are classified as ‘normal’.

*Table S3 (continued) Norms (percentiles) for the self-reported SDQ version for adolescents aged 12 to 17, without distinguishing between genders*

| | | **Scale score** | | | | | | | | | | | | | | | | | | | | |
|---|---|---|---|---|---|---|---|---|---|---|---|---|---|---|---|---|---|---|---|---|---|---|
| **SDQ Scale** | **Age** | **0** | **1** | **2** | **3** | **4** | **5** | **6** | **7** | **8** | **9** | **10** | **11** | **12** | **13** | **14** | **15** | **16** | **17** | **18** | **19** | **20** |
| Externalizing | 12 | 4.1 | 12.3 | 23.1 | 35.4 | 47.7 | 59.3 | 69.5 | 78.0 | 84.7 | 89.9 | 93.6 | 96.2 | 97.8 | 98.9 | 99.5 | 99.8 | 99.9 | 100 | 100 | 100 | 100 |
| | 13 | 4.4 | 12.8 | 23.8 | 36.1 | 48.5 | 59.9 | 70.0 | 78.3 | 85.0 | 90.0 | 93.7 | 96.2 | 97.9 | 98.9 | 99.5 | 99.8 | 99.9 | 100 | 100 | 100 | 100 |
| | 14 | 4.6 | 13.3 | 24.5 | 36.9 | 49.2 | 60.6 | 70.5 | 78.7 | 85.2 | 90.2 | 93.8 | 96.2 | 97.9 | 98.9 | 99.5 | 99.8 | 99.9 | 100 | 100 | 100 | 100 |
| | 15 | 4.9 | 13.8 | 25.3 | 37.7 | 49.9 | 61.2 | 71.0 | 79.1 | 85.5 | 90.3 | 93.9 | 96.3 | 97.9 | 98.9 | 99.5 | 99.8 | 99.9 | 100 | 100 | 100 | 100 |
| | 16 | 5.2 | 14.4 | 26.0 | 38.4 | 50.6 | 61.8 | 71.4 | 79.4 | 85.7 | 90.5 | 93.9 | 96.3 | 97.9 | 98.9 | 99.5 | 99.8 | 99.9 | 100 | 100 | 100 | 100 |
| | 17 | 5.4 | 14.9 | 26.7 | 39.2 | 51.4 | 62.4 | 71.9 | 79.7 | 85.9 | 90.6 | 94.0 | 96.4 | 97.9 | 98.9 | 99.5 | 99.8 | 99.9 | 100 | 100 | 100 | 100 |
| Internalizing | 12 | 19.5 | 36.5 | 50.6 | 62.2 | 71.5 | 78.9 | 84.7 | 89.2 | 92.5 | 94.9 | 96.7 | 97.9 | 98.8 | 99.3 | 99.6 | 99.8 | 99.9 | 100 | 100 | 100 | 100 |
| | 13 | 16.5 | 32.8 | 47.2 | 59.5 | 69.5 | 77.6 | 83.8 | 88.7 | 92.3 | 94.9 | 96.7 | 98.0 | 98.8 | 99.4 | 99.7 | 99.8 | 99.9 | 100 | 100 | 100 | 100 |
| | 14 | 13.7 | 29.1 | 43.7 | 56.5 | 67.3 | 76.0 | 82.8 | 88.1 | 92.0 | 94.8 | 96.7 | 98.0 | 98.9 | 99.4 | 99.7 | 99.9 | 99.9 | 100 | 100 | 100 | 100 |
| | 15 | 11.2 | 25.6 | 40.1 | 53.4 | 64.8 | 74.2 | 81.7 | 87.4 | 91.6 | 94.6 | 96.7 | 98.1 | 98.9 | 99.4 | 99.7 | 99.9 | 100 | 100 | 100 | 100 | 100 |
| | 16 | 9.0 | 22.1 | 36.4 | 50.0 | 62.1 | 72.3 | 80.4 | 86.6 | 91.2 | 94.4 | 96.6 | 98.1 | 99.0 | 99.5 | 99.8 | 99.9 | 100 | 100 | 100 | 100 | 100 |
| | 17 | 7.1 | 18.9 | 32.7 | 46.5 | 59.2 | 70.1 | 78.8 | 85.6 | 90.6 | 94.2 | 96.5 | 98.0 | 99.0 | 99.5 | 99.8 | 99.9 | 100 | 100 | 100 | 100 | 100 |
| | | **0** | **1** | **2** | **3** | **4** | **5** | **6** | **7** | **8** | **9** | **10** | **11** | **12** | **13** | **14** | **15** | **16** | **17** | **18** | **19** | **20** |
| Total | 12 | 0.0 | 2.6 | 6.4 | 11.6 | 18.1 | 25.7 | 34.1 | 43.0 | 51.8 | 60.0 | 67.2 | 73.3 | 78.5 | 82.8 | 86.3 | 89.1 | 91.4 | 93.3 | 94.7 | 95.9 | 96.8 |
| | 13 | 0.0 | 2.6 | 6.3 | 11.4 | 17.8 | 25.4 | 33.7 | 42.4 | 51.2 | 59.4 | 66.6 | 72.8 | 78.0 | 82.4 | 85.9 | 88.8 | 91.2 | 93.0 | 94.5 | 95.7 | 96.7 |
| | 14 | 0.0 | 2.6 | 6.3 | 11.3 | 17.6 | 25.0 | 33.3 | 41.9 | 50.6 | 58.8 | 66.0 | 72.3 | 77.5 | 81.9 | 85.5 | 88.5 | 90.9 | 92.8 | 94.3 | 95.6 | 96.5 |
| | 15 | 0.0 | 2.5 | 6.2 | 11.2 | 17.4 | 24.7 | 32.8 | 41.4 | 50.1 | 58.2 | 65.5 | 71.8 | 77.1 | 81.5 | 85.2 | 88.2 | 90.6 | 92.6 | 94.1 | 95.4 | 96.4 |
| | 16 | 0.0 | 2.5 | 6.1 | 11.0 | 17.2 | 24.4 | 32.4 | 40.9 | 49.5 | 57.7 | 64.9 | 71.2 | 76.6 | 81.1 | 84.8 | 87.8 | 90.3 | 92.3 | 93.9 | 95.2 | 96.2 |
| | 17 | 0.0 | 2.5 | 6.0 | 10.9 | 17.0 | 24.1 | 32.0 | 40.4 | 49.0 | 57.1 | 64.4 | 70.7 | 76.1 | 80.6 | 84.4 | 87.5 | 90.0 | 92.1 | 93.7 | 95.0 | 96.1 |
| | | **21** | **22** | **23** | **24** | **25** | **26** | **27** | **28** | **29** | **30** | **31** | **32** | **33** | **34** | **35** | **36** | **37** | **38** | **39** | **40** | |
| | 12 | 97.5 | 98.1 | 98.5 | 98.9 | 99.1 | 99.3 | 99.5 | 99.6 | 99.7 | 99.8 | 99.8 | 99.9 | 99.9 | 99.9 | 99.9 | 100 | 100 | 100 | 100 | 100 | |
| | 13 | 97.4 | 98.0 | 98.4 | 98.8 | 99.1 | 99.3 | 99.4 | 99.6 | 99.7 | 99.7 | 99.8 | 99.9 | 99.9 | 99.9 | 99.9 | 100 | 100 | 100 | 100 | 100 | |
| | 14 | 97.3 | 97.9 | 98.4 | 98.7 | 99.0 | 99.2 | 99.4 | 99.5 | 99.7 | 99.7 | 99.8 | 99.8 | 99.9 | 99.9 | 99.9 | 99.9 | 100 | 100 | 100 | 100 | |
| | 15 | 97.2 | 97.8 | 98.3 | 98.7 | 99.0 | 99.2 | 99.4 | 99.5 | 99.6 | 99.7 | 99.8 | 99.8 | 99.9 | 99.9 | 99.9 | 99.9 | 100 | 100 | 100 | 100 | |
| | 16 | 97.0 | 97.7 | 98.2 | 98.6 | 98.9 | 99.2 | 99.3 | 99.5 | 99.6 | 99.7 | 99.8 | 99.8 | 99.9 | 99.9 | 99.9 | 99.9 | 100 | 100 | 100 | 100 | |
| | 17 | 96.9 | 97.6 | 98.1 | 98.5 | 98.9 | 99.1 | 99.3 | 99.5 | 99.6 | 99.7 | 99.7 | 99.8 | 99.9 | 99.9 | 99.9 | 99.9 | 99.9 | 100 | 100 | 100 | |

SDQ = Strengths and Difficulties Questionnaire.
Cells containing percentiles ≥ 90 are shaded dark gray and cells containing percentiles from 80 up to 90 light gray, with the corresponding scale scores classified as falling in the ‘abnormal’ and ‘borderline’ range, respectively. The remaining scale scores are classified as ‘normal’.

*Table S4 Norms (percentiles) for the parent-reported SDQ version for female adolescents aged 12 to 17*

| | | **Scale score** | | | | | | | | | | |
|---|---|---|---|---|---|---|---|---|---|---|---|---|
| SDQ Scale | Age | **0** | **1** | **2** | **3** | **4** | **5** | **6** | **7** | **8** | **9** | **10** |
| Emotional | 12 | 32.7 | 52.8 | 67.2 | 77.8 | 85.6 | 91.1 | 95.0 | 97.5 | 99.0 | 99.8 | 100 |
| | 13 | 32.4 | 52.9 | 67.5 | 78.2 | 86.0 | 91.5 | 95.3 | 97.7 | 99.1 | 99.8 | 100 |
| | 14 | 32.2 | 52.9 | 67.8 | 78.6 | 86.4 | 91.9 | 95.6 | 97.9 | 99.2 | 99.8 | 100 |
| | 15 | 31.9 | 52.9 | 68.1 | 79.0 | 86.9 | 92.3 | 95.9 | 98.1 | 99.3 | 99.8 | 100 |
| | 16 | 31.7 | 53.0 | 68.3 | 79.4 | 87.3 | 92.7 | 96.1 | 98.2 | 99.4 | 99.9 | 100 |
| | 17 | 31.5 | 53.1 | 68.7 | 79.8 | 87.7 | 93.0 | 96.4 | 98.4 | 99.4 | 99.9 | 100 |
| Conduct | 12 | 45.7 | 74.5 | 89.3 | 95.9 | 98.6 | 99.6 | 99.9 | 100 | 100 | 100 | 100 |
| | 13 | 47.0 | 75.0 | 89.2 | 95.8 | 98.5 | 99.5 | 99.9 | 100 | 100 | 100 | 100 |
| | 14 | 48.4 | 75.4 | 89.1 | 95.6 | 98.4 | 99.5 | 99.9 | 100 | 100 | 100 | 100 |
| | 15 | 49.8 | 75.8 | 89.1 | 95.4 | 98.2 | 99.4 | 99.8 | 100 | 100 | 100 | 100 |
| | 16 | 51.2 | 76.3 | 89.0 | 95.2 | 98.1 | 99.3 | 99.8 | 99.9 | 100 | 100 | 100 |
| | 17 | 52.6 | 76.7 | 88.9 | 95.0 | 97.9 | 99.2 | 99.7 | 99.9 | 100 | 100 | 100 |
| Hyperactivity | 12 | 29.8 | 47.6 | 60.8 | 71.2 | 79.4 | 85.9 | 91.0 | 94.8 | 97.5 | 99.2 | 100 |
| | 13 | 30.4 | 49.1 | 62.8 | 73.4 | 81.6 | 87.9 | 92.6 | 96.0 | 98.2 | 99.5 | 100 |
| | 14 | 31.1 | 50.6 | 64.9 | 75.7 | 83.8 | 89.8 | 94.1 | 96.9 | 98.7 | 99.7 | 100 |
| | 15 | 31.8 | 52.3 | 67.1 | 77.9 | 85.8 | 91.5 | 95.3 | 97.7 | 99.1 | 99.8 | 100 |
| | 16 | 32.7 | 54.1 | 69.3 | 80.1 | 87.8 | 93.0 | 96.3 | 98.3 | 99.4 | 99.9 | 100 |
| | 17 | 33.7 | 55.9 | 71.5 | 82.3 | 89.6 | 94.3 | 97.2 | 98.8 | 99.6 | 99.9 | 100 |
| Social | 12 | 48.2 | 68.2 | 80.1 | 87.7 | 92.7 | 95.9 | 97.9 | 99.1 | 99.7 | 99.9 | 100 |
| | 13 | 46.5 | 67.2 | 79.7 | 87.7 | 92.8 | 96.1 | 98.1 | 99.2 | 99.7 | 99.9 | 100 |
| | 14 | 44.8 | 66.2 | 79.3 | 87.6 | 93.0 | 96.2 | 98.2 | 99.2 | 99.8 | 100 | 100 |
| | 15 | 43.1 | 65.2 | 78.9 | 87.6 | 93.1 | 96.4 | 98.3 | 99.3 | 99.8 | 100 | 100 |
| | 16 | 41.4 | 64.2 | 78.5 | 87.6 | 93.2 | 96.5 | 98.4 | 99.4 | 99.8 | 100 | 100 |
| | 17 | 39.8 | 63.2 | 78.1 | 87.5 | 93.3 | 96.7 | 98.5 | 99.5 | 99.8 | 100 | 100 |
| prosocial | 12 | 100 | 99.6 | 98.9 | 97.5 | 95.1 | 91.4 | 85.7 | 77.1 | 64.2 | 43.6 | 0.0 |
| | 13 | 100 | 99.7 | 99.0 | 97.7 | 95.3 | 91.4 | 85.3 | 76.1 | 62.1 | 40.5 | 0.0 |
| | 14 | 100 | 99.7 | 99.1 | 97.8 | 95.4 | 91.4 | 84.9 | 74.9 | 60.0 | 37.4 | 0.0 |
| | 15 | 100 | 99.8 | 99.2 | 98.0 | 95.6 | 91.4 | 84.5 | 73.8 | 57.8 | 34.4 | 0.0 |
| | 16 | 100 | 99.8 | 99.3 | 98.1 | 95.7 | 91.3 | 84.0 | 72.5 | 55.5 | 31.5 | 0.0 |
| | 17 | 100 | 99.8 | 99.4 | 98.2 | 95.8 | 91.2 | 83.4 | 71.1 | 53.1 | 28.7 | 0.0 |

SDQ = Strengths and Difficulties Questionnaire.
Cells containing percentiles ≥ 90 are shaded dark gray and cells containing percentiles from 80 up to 90 light gray, with the corresponding scale scores classified as falling in the 'abnormal' and 'borderline' range, respectively. The remaining scale scores are classified as 'normal'.

*Table S4 (continued) Norms (percentiles) for the parent-reported SDQ version for female adolescents aged 12 to 17*

| | | Scale score | | | | | | | | | | | | | | | | | | | | |
|---|---|---|---|---|---|---|---|---|---|---|---|---|---|---|---|---|---|---|---|---|---|---|
| **SDQ Scale** | **Age** | **0** | **1** | **2** | **3** | **4** | **5** | **6** | **7** | **8** | **9** | **10** | **11** | **12** | **13** | **14** | **15** | **16** | **17** | **18** | **19** | **20** |
| Externalizing | 12 | 20.2 | 36.6 | 50.0 | 61.1 | 70.1 | 77.3 | 83.1 | 87.6 | 91.1 | 93.8 | 95.8 | 97.3 | 98.3 | 99.0 | 99.4 | 99.7 | 99.9 | 99.9 | 100 | 100 | 100 |
| | 13 | 21.1 | 38.1 | 51.9 | 63.0 | 71.9 | 79.0 | 84.6 | 88.9 | 92.2 | 94.6 | 96.4 | 97.7 | 98.6 | 99.2 | 99.6 | 99.8 | 99.9 | 100 | 100 | 100 | 100 |
| | 14 | 22.1 | 39.7 | 53.7 | 64.9 | 73.7 | 80.6 | 86.0 | 90.1 | 93.1 | 95.4 | 97.0 | 98.1 | 98.9 | 99.3 | 99.7 | 99.8 | 99.9 | 100 | 100 | 100 | 100 |
| | 15 | 23.2 | 41.3 | 55.5 | 66.7 | 75.5 | 82.2 | 87.3 | 91.2 | 94.0 | 96.0 | 97.5 | 98.4 | 99.1 | 99.5 | 99.7 | 99.9 | 99.9 | 100 | 100 | 100 | 100 |
| | 16 | 24.3 | 42.9 | 57.4 | 68.6 | 77.2 | 83.7 | 88.6 | 92.2 | 94.8 | 96.6 | 97.9 | 98.7 | 99.3 | 99.6 | 99.8 | 99.9 | 100 | 100 | 100 | 100 | 100 |
| | 17 | 25.4 | 44.6 | 59.2 | 70.4 | 78.8 | 85.1 | 89.7 | 93.1 | 95.5 | 97.1 | 98.2 | 99.0 | 99.4 | 99.7 | 99.8 | 99.9 | 100 | 100 | 100 | 100 | 100 |
| Internalizing | 12 | 22.1 | 38.6 | 51.7 | 62.3 | 70.9 | 77.8 | 83.3 | 87.7 | 91.1 | 93.7 | 95.6 | 97.1 | 98.1 | 98.9 | 99.4 | 99.7 | 99.8 | 99.9 | 100 | 100 | 100 |
| | 13 | 21.3 | 37.9 | 51.3 | 62.1 | 70.8 | 77.8 | 83.4 | 87.8 | 91.3 | 93.9 | 95.8 | 97.2 | 98.3 | 98.9 | 99.4 | 99.7 | 99.9 | 99.9 | 100 | 100 | 100 |
| | 14 | 20.7 | 37.2 | 50.8 | 61.8 | 70.7 | 77.9 | 83.6 | 88.0 | 91.4 | 94.0 | 96.0 | 97.4 | 98.4 | 99.0 | 99.5 | 99.7 | 99.9 | 100 | 100 | 100 | 100 |
| | 15 | 20.0 | 36.6 | 50.3 | 61.5 | 70.6 | 77.9 | 83.7 | 88.2 | 91.6 | 94.2 | 96.1 | 97.5 | 98.5 | 99.1 | 99.5 | 99.8 | 99.9 | 100 | 100 | 100 | 100 |
| | 16 | 19.3 | 35.9 | 49.8 | 61.2 | 70.5 | 78.0 | 83.8 | 88.4 | 91.8 | 94.4 | 96.3 | 97.6 | 98.6 | 99.2 | 99.6 | 99.8 | 99.9 | 100 | 100 | 100 | 100 |
| | 17 | 18.7 | 35.3 | 49.3 | 61.0 | 70.4 | 78.0 | 83.9 | 88.5 | 92.0 | 94.6 | 96.5 | 97.8 | 98.7 | 99.2 | 99.6 | 99.8 | 99.9 | 100 | 100 | 100 | 100 |
| | | **0** | **1** | **2** | **3** | **4** | **5** | **6** | **7** | **8** | **9** | **10** | **11** | **12** | **13** | **14** | **15** | **16** | **17** | **18** | **19** | **20** |
| Total | 12 | 0.1 | 10.8 | 20.3 | 29.9 | 39.4 | 48.3 | 56.4 | 63.3 | 69.1 | 74.0 | 78.1 | 81.5 | 84.4 | 86.8 | 88.8 | 90.5 | 92.0 | 93.2 | 94.2 | 95.1 | 95.8 |
| | 13 | 0.1 | 11.0 | 20.7 | 30.6 | 40.2 | 49.3 | 57.4 | 64.3 | 70.1 | 74.9 | 79.0 | 82.3 | 85.1 | 87.5 | 89.4 | 91.1 | 92.4 | 93.6 | 94.6 | 95.4 | 96.1 |
| | 14 | 0.1 | 11.2 | 21.2 | 31.3 | 41.1 | 50.4 | 58.5 | 65.4 | 71.1 | 75.9 | 79.8 | 83.1 | 85.9 | 88.1 | 90.0 | 91.6 | 92.9 | 94.0 | 94.9 | 95.7 | 96.4 |
| | 15 | 0.1 | 11.5 | 21.7 | 32.0 | 42.1 | 51.4 | 59.6 | 66.4 | 72.1 | 76.8 | 80.7 | 83.9 | 86.6 | 88.8 | 90.6 | 92.1 | 93.4 | 94.4 | 95.3 | 96.0 | 96.7 |
| | 16 | 0.1 | 11.7 | 22.2 | 32.8 | 43.1 | 52.5 | 60.7 | 67.5 | 73.1 | 77.8 | 81.6 | 84.7 | 87.3 | 89.4 | 91.2 | 92.6 | 93.8 | 94.8 | 95.7 | 96.4 | 96.9 |
| | 17 | 0.1 | 12.0 | 22.8 | 33.6 | 44.1 | 53.7 | 61.9 | 68.6 | 74.2 | 78.7 | 82.4 | 85.5 | 88.0 | 90.0 | 91.7 | 93.1 | 94.3 | 95.2 | 96.0 | 96.6 | 97.2 |
| | | **21** | **22** | **23** | **24** | **25** | **26** | **27** | **28** | **29** | **30** | **31** | **32** | **33** | **34** | **35** | **36** | **37** | **38** | **39** | **40** | |
| | 12 | 96.4 | 96.9 | 97.4 | 97.7 | 98.1 | 98.3 | 98.6 | 98.8 | 98.9 | 99.1 | 99.2 | 99.3 | 99.4 | 99.5 | 99.6 | 99.6 | 99.7 | 99.7 | 99.8 | 100 | |
| | 13 | 96.7 | 97.2 | 97.6 | 97.9 | 98.2 | 98.5 | 98.7 | 98.9 | 99.0 | 99.2 | 99.3 | 99.4 | 99.5 | 99.5 | 99.6 | 99.7 | 99.7 | 99.7 | 99.8 | 100 | |
| | 14 | 96.9 | 97.4 | 97.8 | 98.1 | 98.4 | 98.6 | 98.8 | 99.0 | 99.1 | 99.3 | 99.4 | 99.5 | 99.5 | 99.6 | 99.7 | 99.7 | 99.7 | 99.8 | 99.8 | 100 | |
| | 15 | 97.2 | 97.6 | 98.0 | 98.3 | 98.5 | 98.8 | 98.9 | 99.1 | 99.2 | 99.3 | 99.4 | 99.5 | 99.6 | 99.6 | 99.7 | 99.7 | 99.8 | 99.8 | 99.9 | 100 | |
| | 16 | 97.4 | 97.8 | 98.2 | 98.4 | 98.7 | 98.9 | 99.0 | 99.2 | 99.3 | 99.4 | 99.5 | 99.6 | 99.6 | 99.7 | 99.7 | 99.8 | 99.8 | 99.8 | 99.9 | 100 | |
| | 17 | 97.6 | 98.0 | 98.3 | 98.6 | 98.8 | 99.0 | 99.1 | 99.3 | 99.4 | 99.5 | 99.6 | 99.6 | 99.7 | 99.7 | 99.8 | 99.8 | 99.8 | 99.9 | 99.9 | 100 | |

SDQ = Strengths and Difficulties Questionnaire.
Cells containing percentiles ≥ 90 are shaded dark gray and cells containing percentiles from 80 up to 90 light gray, with the corresponding scale scores classified as falling in the ‘abnormal’ and ‘borderline’ range, respectively. The remaining scale scores are classified as ‘normal’.

*Table S5 Norms (percentiles) for the parent-reported SDQ version for male adolescents aged 12 to 17*

| | | **Scale score** | | | | | | | | | | |
|---|---|---|---|---|---|---|---|---|---|---|---|---|
| SDQ Scale | Age | **0** | **1** | **2** | **3** | **4** | **5** | **6** | **7** | **8** | **9** | **10** |
| Emotional | 12 | 39.9 | 58.1 | 70.3 | 79.1 | 85.6 | 90.6 | 94.2 | 96.8 | 98.5 | 99.6 | 100 |
| | 13 | 39.6 | 58.1 | 70.5 | 79.4 | 86.0 | 91.0 | 94.5 | 97.0 | 98.7 | 99.6 | 100 |
| | 14 | 39.3 | 58.2 | 70.7 | 79.8 | 86.4 | 91.3 | 94.8 | 97.3 | 98.8 | 99.7 | 100 |
| | 15 | 39.0 | 58.2 | 71.0 | 80.1 | 86.8 | 91.7 | 95.1 | 97.5 | 98.9 | 99.7 | 100 |
| | 16 | 38.7 | 58.2 | 71.2 | 80.5 | 87.2 | 92.1 | 95.4 | 97.7 | 99.0 | 99.7 | 100 |
| | 17 | 38.4 | 58.2 | 71.5 | 80.9 | 87.6 | 92.4 | 95.7 | 97.9 | 99.1 | 99.8 | 100 |
| Conduct | 12 | 47.3 | 71.4 | 84.9 | 92.4 | 96.4 | 98.5 | 99.4 | 99.8 | 100 | 100 | 100 |
| | 13 | 48.9 | 72.0 | 84.9 | 92.1 | 96.2 | 98.3 | 99.3 | 99.8 | 99.9 | 100 | 100 |
| | 14 | 50.6 | 72.7 | 84.9 | 91.9 | 95.9 | 98.1 | 99.2 | 99.7 | 99.9 | 100 | 100 |
| | 15 | 52.3 | 73.3 | 84.9 | 91.7 | 95.6 | 97.9 | 99.1 | 99.7 | 99.9 | 100 | 100 |
| | 16 | 54.0 | 74.0 | 85.0 | 91.5 | 95.4 | 97.6 | 98.9 | 99.6 | 99.9 | 100 | 100 |
| | 17 | 55.7 | 74.7 | 85.0 | 91.3 | 95.1 | 97.4 | 98.8 | 99.5 | 99.8 | 100 | 100 |
| Hyperactivity | 12 | 13.5 | 27.2 | 40.3 | 52.4 | 63.5 | 73.3 | 81.8 | 88.8 | 94.2 | 98.0 | 100 |
| | 13 | 13.7 | 28.2 | 42.1 | 54.9 | 66.4 | 76.2 | 84.4 | 90.9 | 95.6 | 98.6 | 100 |
| | 14 | 14.1 | 29.4 | 44.1 | 57.6 | 69.3 | 79.1 | 86.9 | 92.8 | 96.7 | 99.1 | 100 |
| | 15 | 14.5 | 30.7 | 46.3 | 60.3 | 72.2 | 81.8 | 89.2 | 94.4 | 97.6 | 99.4 | 100 |
| | 16 | 15.0 | 32.2 | 48.6 | 63.1 | 75.1 | 84.4 | 91.2 | 95.7 | 98.3 | 99.6 | 100 |
| | 17 | 15.6 | 33.8 | 51.1 | 66.0 | 77.9 | 86.8 | 92.9 | 96.8 | 98.8 | 99.8 | 100 |
| Social | 12 | 33.7 | 56.1 | 71.8 | 82.6 | 89.8 | 94.5 | 97.3 | 98.9 | 99.6 | 99.9 | 100 |
| | 13 | 32.0 | 54.9 | 71.1 | 82.4 | 89.9 | 94.6 | 97.5 | 99.0 | 99.7 | 99.9 | 100 |
| | 14 | 30.4 | 53.6 | 70.5 | 82.2 | 89.9 | 94.8 | 97.6 | 99.1 | 99.7 | 99.9 | 100 |
| | 15 | 28.8 | 52.4 | 69.8 | 82.0 | 90.0 | 94.9 | 97.7 | 99.1 | 99.7 | 100 | 100 |
| | 16 | 27.3 | 51.1 | 69.1 | 81.8 | 90.0 | 95.0 | 97.8 | 99.2 | 99.8 | 100 | 100 |
| | 17 | 25.8 | 49.8 | 68.4 | 81.5 | 90.1 | 95.2 | 97.9 | 99.3 | 99.8 | 100 | 100 |
| prosocial | 12 | 100 | 99.8 | 99.3 | 98.0 | 95.4 | 90.8 | 82.9 | 70.8 | 53.1 | 29.1 | 0.0 |
| | 13 | 100 | 99.8 | 99.3 | 98.1 | 95.5 | 90.6 | 82.3 | 69.3 | 50.7 | 26.3 | 0.0 |
| | 14 | 100 | 99.9 | 99.4 | 98.2 | 95.6 | 90.4 | 81.6 | 67.8 | 48.2 | 23.7 | 0.0 |
| | 15 | 100 | 99.9 | 99.5 | 98.3 | 95.6 | 90.2 | 80.8 | 66.1 | 45.6 | 21.3 | 0.0 |
| | 16 | 100 | 99.9 | 99.5 | 98.3 | 95.5 | 89.9 | 79.9 | 64.3 | 43.0 | 19.0 | 0.0 |
| | 17 | 100 | 99.9 | 99.5 | 98.4 | 95.5 | 89.6 | 78.9 | 62.4 | 40.4 | 16.8 | 0.0 |

SDQ = Strengths and Difficulties Questionnaire.
Cells containing percentiles ≥ 90 are shaded dark gray and cells containing percentiles from 80 up to 90 light gray, with the corresponding scale scores classified as falling in the 'abnormal' and 'borderline' range, respectively. The remaining scale scores are classified as 'normal'.

*Table S5 (continued) Norms (percentiles) for the parent-reported SDQ version for male adolescents aged 12 to 17*

| | | Scale score | | | | | | | | | | | | | | | | | | | | |
|---|---|---|---|---|---|---|---|---|---|---|---|---|---|---|---|---|---|---|---|---|---|---|
| **SDQ Scale** | **Age** | **0** | **1** | **2** | **3** | **4** | **5** | **6** | **7** | **8** | **9** | **10** | **11** | **12** | **13** | **14** | **15** | **16** | **17** | **18** | **19** | **20** |
| Externalizing | 12 | 10.1 | 21.8 | 33.5 | 44.5 | 54.5 | 63.4 | 71.2 | 77.7 | 83.2 | 87.6 | 91.1 | 93.9 | 95.9 | 97.4 | 98.5 | 99.2 | 99.6 | 99.8 | 99.9 | 100 | 100 |
| | 13 | 10.7 | 23.0 | 35.2 | 46.5 | 56.7 | 65.6 | 73.2 | 79.6 | 84.8 | 89.0 | 92.3 | 94.8 | 96.6 | 97.9 | 98.8 | 99.4 | 99.7 | 99.9 | 100 | 100 | 100 |
| | 14 | 11.4 | 24.3 | 36.9 | 48.6 | 58.9 | 67.8 | 75.3 | 81.5 | 86.4 | 90.3 | 93.3 | 95.6 | 97.2 | 98.3 | 99.0 | 99.5 | 99.8 | 99.9 | 100 | 100 | 100 |
| | 15 | 12.1 | 25.7 | 38.8 | 50.7 | 61.1 | 69.9 | 77.3 | 83.2 | 87.9 | 91.6 | 94.3 | 96.3 | 97.7 | 98.6 | 99.3 | 99.6 | 99.8 | 99.9 | 100 | 100 | 100 |
| | 16 | 12.9 | 27.1 | 40.6 | 52.8 | 63.2 | 72.0 | 79.2 | 84.9 | 89.3 | 92.7 | 95.1 | 96.9 | 98.1 | 98.9 | 99.4 | 99.7 | 99.9 | 100 | 100 | 100 | 100 |
| | 17 | 13.7 | 28.6 | 42.6 | 54.9 | 65.4 | 74.1 | 81.0 | 86.4 | 90.6 | 93.7 | 95.9 | 97.4 | 98.5 | 99.2 | 99.6 | 99.8 | 99.9 | 100 | 100 | 100 | 100 |
| Internalizing | 12 | 19.5 | 35.0 | 47.9 | 58.6 | 67.4 | 74.7 | 80.6 | 85.4 | 89.2 | 92.2 | 94.5 | 96.3 | 97.6 | 98.5 | 99.1 | 99.5 | 99.8 | 99.9 | 100 | 100 | 100 |
| | 13 | 18.8 | 34.3 | 47.4 | 58.2 | 67.2 | 74.7 | 80.7 | 85.6 | 89.4 | 92.4 | 94.7 | 96.4 | 97.7 | 98.6 | 99.2 | 99.6 | 99.8 | 99.9 | 100 | 100 | 100 |
| | 14 | 18.1 | 33.7 | 46.8 | 57.9 | 67.1 | 74.7 | 80.8 | 85.7 | 89.6 | 92.6 | 94.9 | 96.6 | 97.8 | 98.7 | 99.2 | 99.6 | 99.8 | 99.9 | 100 | 100 | 100 |
| | 15 | 17.5 | 33.0 | 46.3 | 57.6 | 66.9 | 74.7 | 80.9 | 85.9 | 89.8 | 92.8 | 95.1 | 96.8 | 97.9 | 98.8 | 99.3 | 99.6 | 99.8 | 99.9 | 100 | 100 | 100 |
| | 16 | 16.8 | 32.3 | 45.8 | 57.2 | 66.8 | 74.7 | 81.0 | 86.1 | 90.0 | 93.0 | 95.3 | 96.9 | 98.1 | 98.8 | 99.4 | 99.7 | 99.9 | 99.9 | 100 | 100 | 100 |
| | 17 | 16.2 | 31.6 | 45.3 | 56.9 | 66.7 | 74.7 | 81.1 | 86.2 | 90.2 | 93.2 | 95.4 | 97.0 | 98.2 | 98.9 | 99.4 | 99.7 | 99.9 | 100 | 100 | 100 | 100 |
| | | **0** | **1** | **2** | **3** | **4** | **5** | **6** | **7** | **8** | **9** | **10** | **11** | **12** | **13** | **14** | **15** | **16** | **17** | **18** | **19** | **20** |
| Total | 12 | 0.0 | 7.4 | 14.3 | 21.6 | 29.2 | 36.9 | 44.5 | 51.7 | 58.3 | 64.0 | 69.0 | 73.4 | 77.1 | 80.4 | 83.2 | 85.5 | 87.6 | 89.3 | 90.8 | 92.1 | 93.2 |
| | 13 | 0.0 | 7.5 | 14.5 | 22.0 | 29.8 | 37.6 | 45.3 | 52.6 | 59.1 | 64.9 | 69.9 | 74.2 | 77.9 | 81.1 | 83.8 | 86.1 | 88.1 | 89.8 | 91.3 | 92.5 | 93.6 |
| | 14 | 0.0 | 7.6 | 14.8 | 22.4 | 30.4 | 38.3 | 46.1 | 53.5 | 60.0 | 65.8 | 70.7 | 75.0 | 78.6 | 81.8 | 84.4 | 86.7 | 88.6 | 90.3 | 91.7 | 92.9 | 93.9 |
| | 15 | 0.0 | 7.8 | 15.1 | 22.9 | 31.0 | 39.1 | 47.0 | 54.4 | 60.9 | 66.6 | 71.6 | 75.8 | 79.4 | 82.4 | 85.1 | 87.3 | 89.2 | 90.8 | 92.1 | 93.3 | 94.3 |
| | 16 | 0.0 | 7.9 | 15.3 | 23.3 | 31.6 | 39.8 | 47.9 | 55.3 | 61.9 | 67.5 | 72.4 | 76.6 | 80.1 | 83.1 | 85.7 | 87.8 | 89.7 | 91.2 | 92.6 | 93.7 | 94.6 |
| | 17 | 0.0 | 8.0 | 15.6 | 23.8 | 32.2 | 40.6 | 48.8 | 56.2 | 62.8 | 68.4 | 73.3 | 77.4 | 80.9 | 83.8 | 86.3 | 88.4 | 90.2 | 91.7 | 93.0 | 94.0 | 94.9 |
| | | **21** | **22** | **23** | **24** | **25** | **26** | **27** | **28** | **29** | **30** | **31** | **32** | **33** | **34** | **35** | **36** | **37** | **38** | **39** | **40** | |
| | 12 | 94.2 | 95.0 | 95.7 | 96.3 | 96.8 | 97.2 | 97.6 | 97.9 | 98.2 | 98.5 | 98.7 | 98.9 | 99.0 | 99.1 | 99.3 | 99.4 | 99.4 | 99.5 | 99.7 | 100 | |
| | 13 | 94.5 | 95.3 | 95.9 | 96.5 | 97.0 | 97.4 | 97.8 | 98.1 | 98.4 | 98.6 | 98.8 | 98.9 | 99.1 | 99.2 | 99.3 | 99.4 | 99.5 | 99.6 | 99.7 | 100 | |
| | 14 | 94.8 | 95.6 | 96.2 | 96.7 | 97.2 | 97.6 | 97.9 | 98.2 | 98.5 | 98.7 | 98.9 | 99.0 | 99.2 | 99.3 | 99.4 | 99.5 | 99.5 | 99.6 | 99.8 | 100 | |
| | 15 | 95.1 | 95.8 | 96.4 | 97.0 | 97.4 | 97.8 | 98.1 | 98.4 | 98.6 | 98.8 | 99.0 | 99.1 | 99.2 | 99.3 | 99.4 | 99.5 | 99.6 | 99.6 | 99.8 | 100 | |
| | 16 | 95.4 | 96.1 | 96.7 | 97.2 | 97.6 | 97.9 | 98.2 | 98.5 | 98.7 | 98.9 | 99.1 | 99.2 | 99.3 | 99.4 | 99.5 | 99.6 | 99.6 | 99.7 | 99.8 | 100 | |
| | 17 | 95.7 | 96.4 | 96.9 | 97.4 | 97.8 | 98.1 | 98.4 | 98.6 | 98.8 | 99.0 | 99.1 | 99.3 | 99.4 | 99.5 | 99.5 | 99.6 | 99.7 | 99.8 | 99.9 | 100 | |

SDQ = Strengths and Difficulties Questionnaire.
Cells containing percentiles ≥ 90 are shaded dark gray and cells containing percentiles from 80 up to 90 light gray, with the corresponding scale scores classified as falling in the 'abnormal' and 'borderline' range, respectively. The remaining scale scores are classified as 'normal'.

*Table S6 Norms (percentiles) for the parent-reported SDQ version for adolescents aged 12 to 17, without distinguishing between genders*

| | | Scale score | | | | | | | | | | |
|---|---|---|---|---|---|---|---|---|---|---|---|---|
| SDQ Scale | Age | **0** | **1** | **2** | **3** | **4** | **5** | **6** | **7** | **8** | **9** | **10** |
| Emotional | 12 | 36.1 | 55.1 | 68.2 | 77.9 | 85.1 | 90.4 | 94.3 | 96.9 | 98.7 | 99.6 | 100 |
| | 13 | 35.9 | 55.3 | 68.7 | 78.5 | 85.7 | 91.0 | 94.7 | 97.3 | 98.8 | 99.7 | 100 |
| | 14 | 35.7 | 55.5 | 69.2 | 79.1 | 86.3 | 91.5 | 95.2 | 97.6 | 99.0 | 99.7 | 100 |
| | 15 | 35.6 | 55.8 | 69.7 | 79.7 | 87.0 | 92.1 | 95.6 | 97.8 | 99.1 | 99.8 | 100 |
| | 16 | 35.4 | 56.0 | 70.2 | 80.4 | 87.6 | 92.6 | 96.0 | 98.1 | 99.3 | 99.8 | 100 |
| | 17 | 35.3 | 56.3 | 70.7 | 81.0 | 88.2 | 93.1 | 96.3 | 98.3 | 99.4 | 99.9 | 100 |
| Conduct | 12 | 46.5 | 71.6 | 85.6 | 93.1 | 96.9 | 98.8 | 99.6 | 99.9 | 100 | 100 | 100 |
| | 13 | 48.1 | 72.8 | 86.2 | 93.4 | 97.0 | 98.8 | 99.6 | 99.9 | 100 | 100 | 100 |
| | 14 | 49.7 | 73.9 | 86.8 | 93.6 | 97.2 | 98.8 | 99.6 | 99.9 | 100 | 100 | 100 |
| | 15 | 51.3 | 74.9 | 87.3 | 93.9 | 97.3 | 98.9 | 99.6 | 99.9 | 100 | 100 | 100 |
| | 16 | 52.9 | 75.9 | 87.9 | 94.1 | 97.4 | 98.9 | 99.6 | 99.9 | 100 | 100 | 100 |
| | 17 | 54.4 | 76.9 | 88.4 | 94.4 | 97.4 | 98.9 | 99.6 | 99.9 | 100 | 100 | 100 |
| Hyperactivity | 12 | 20.7 | 36.5 | 49.7 | 60.9 | 70.6 | 78.7 | 85.6 | 91.1 | 95.4 | 98.4 | 100 |
| | 13 | 21.4 | 38.1 | 52.1 | 63.8 | 73.6 | 81.6 | 88.1 | 93.1 | 96.7 | 98.9 | 100 |
| | 14 | 22.2 | 39.9 | 54.6 | 66.7 | 76.6 | 84.4 | 90.4 | 94.7 | 97.6 | 99.3 | 100 |
| | 15 | 23.0 | 41.9 | 57.3 | 69.7 | 79.4 | 86.9 | 92.3 | 96.1 | 98.4 | 99.6 | 100 |
| | 16 | 24.0 | 44.0 | 60.0 | 72.6 | 82.2 | 89.2 | 94.0 | 97.1 | 98.9 | 99.7 | 100 |
| | 17 | 25.1 | 46.2 | 62.9 | 75.6 | 84.8 | 91.3 | 95.5 | 98.0 | 99.3 | 99.8 | 100 |
| Social | 12 | 39.8 | 61.4 | 75.4 | 84.8 | 91.0 | 95.1 | 97.5 | 98.9 | 99.6 | 99.9 | 100 |
| | 13 | 38.6 | 60.7 | 75.2 | 84.8 | 91.2 | 95.2 | 97.7 | 99.0 | 99.7 | 99.9 | 100 |
| | 14 | 37.4 | 60.0 | 74.9 | 84.9 | 91.4 | 95.4 | 97.8 | 99.1 | 99.7 | 99.9 | 100 |
| | 15 | 36.3 | 59.3 | 74.7 | 84.9 | 91.6 | 95.6 | 98.0 | 99.2 | 99.8 | 100 | 100 |
| | 16 | 35.1 | 58.6 | 74.5 | 85.0 | 91.7 | 95.8 | 98.1 | 99.3 | 99.8 | 100 | 100 |
| | 17 | 34.0 | 57.9 | 74.2 | 85.1 | 91.9 | 96.0 | 98.2 | 99.3 | 99.8 | 100 | 100 |
| prosocial | 12 | 100 | 99.7 | 99.1 | 97.8 | 95.3 | 91.0 | 84.2 | 73.7 | 58.2 | 35.4 | 0.0 |
| | 13 | 100 | 99.8 | 99.2 | 97.9 | 95.4 | 90.9 | 83.7 | 72.6 | 56.2 | 32.8 | 0.0 |
| | 14 | 100 | 99.8 | 99.3 | 98.0 | 95.4 | 90.8 | 83.2 | 71.5 | 54.2 | 30.3 | 0.0 |
| | 15 | 100 | 99.8 | 99.3 | 98.1 | 95.5 | 90.7 | 82.7 | 70.2 | 52.1 | 27.9 | 0.0 |
| | 16 | 100 | 99.8 | 99.4 | 98.2 | 95.5 | 90.6 | 82.1 | 68.9 | 49.9 | 25.6 | 0.0 |
| | 17 | 100 | 99.9 | 99.4 | 98.2 | 95.6 | 90.4 | 81.5 | 67.5 | 47.8 | 23.3 | 0.0 |

SDQ = Strengths and Difficulties Questionnaire.
Cells containing percentiles ≥ 90 are shaded dark gray and cells containing percentiles from 80 up to 90 light gray, with the corresponding scale scores classified as falling in the 'abnormal' and 'borderline' range, respectively. The remaining scale scores are classified as 'normal'.

*Table S6 (continued) Norms (percentiles) for the parent-reported SDQ version for adolescents aged 12 to 17, without distinguishing between genders*

| | | **Scale score** | | | | | | | | | | | | | | | | | | | | |
|---|---|---|---|---|---|---|---|---|---|---|---|---|---|---|---|---|---|---|---|---|---|---|
| **SDQ Scale** | **Age** | **0** | **1** | **2** | **3** | **4** | **5** | **6** | **7** | **8** | **9** | **10** | **11** | **12** | **13** | **14** | **15** | **16** | **17** | **18** | **19** | **20** |
| Externalizing | 12 | 14.8 | 28.7 | 41.1 | 52.0 | 61.4 | 69.4 | 76.2 | 81.7 | 86.3 | 89.9 | 92.8 | 95.0 | 96.7 | 97.9 | 98.7 | 99.3 | 99.7 | 99.9 | 100 | 100 | 100 |
| | 13 | 15.7 | 30.2 | 43.2 | 54.3 | 63.8 | 71.8 | 78.4 | 83.8 | 88.1 | 91.4 | 94.0 | 96.0 | 97.4 | 98.4 | 99.1 | 99.5 | 99.8 | 99.9 | 100 | 100 | 100 |
| | 14 | 16.5 | 31.8 | 45.2 | 56.7 | 66.3 | 74.2 | 80.6 | 85.7 | 89.7 | 92.8 | 95.1 | 96.8 | 98.0 | 98.8 | 99.3 | 99.7 | 99.8 | 99.9 | 100 | 100 | 100 |
| | 15 | 17.5 | 33.5 | 47.4 | 59.0 | 68.7 | 76.4 | 82.6 | 87.5 | 91.2 | 94.0 | 96.0 | 97.4 | 98.4 | 99.1 | 99.5 | 99.8 | 99.9 | 100 | 100 | 100 | 100 |
| | 16 | 18.5 | 35.3 | 49.6 | 61.4 | 71.0 | 78.6 | 84.6 | 89.1 | 92.5 | 95.0 | 96.8 | 98.0 | 98.8 | 99.3 | 99.7 | 99.8 | 99.9 | 100 | 100 | 100 | 100 |
| | 17 | 19.6 | 37.1 | 51.8 | 63.8 | 73.3 | 80.7 | 86.4 | 90.6 | 93.7 | 95.9 | 97.4 | 98.5 | 99.1 | 99.5 | 99.8 | 99.9 | 100 | 100 | 100 | 100 | 100 |
| Internalizing | 12 | 20.6 | 36.5 | 49.4 | 60.0 | 68.7 | 75.8 | 81.6 | 86.2 | 89.9 | 92.7 | 94.9 | 96.5 | 97.7 | 98.6 | 99.2 | 99.6 | 99.8 | 99.9 | 100 | 100 | 100 |
| | 13 | 20.0 | 36.0 | 49.1 | 59.9 | 68.8 | 76.0 | 81.9 | 86.5 | 90.2 | 93.0 | 95.2 | 96.8 | 97.9 | 98.7 | 99.3 | 99.6 | 99.8 | 99.9 | 100 | 100 | 100 |
| | 14 | 19.4 | 35.4 | 48.7 | 59.8 | 68.8 | 76.2 | 82.1 | 86.8 | 90.5 | 93.3 | 95.4 | 97.0 | 98.1 | 98.8 | 99.3 | 99.7 | 99.8 | 99.9 | 100 | 100 | 100 |
| | 15 | 18.8 | 34.9 | 48.4 | 59.7 | 68.9 | 76.4 | 82.4 | 87.1 | 90.8 | 93.6 | 95.7 | 97.2 | 98.2 | 98.9 | 99.4 | 99.7 | 99.9 | 99.9 | 100 | 100 | 100 |
| | 16 | 18.2 | 34.3 | 48.1 | 59.5 | 69.0 | 76.6 | 82.7 | 87.4 | 91.1 | 93.9 | 95.9 | 97.4 | 98.4 | 99.1 | 99.5 | 99.7 | 99.9 | 100 | 100 | 100 | 100 |
| | 17 | 17.6 | 33.8 | 47.7 | 59.4 | 69.1 | 76.8 | 83.0 | 87.8 | 91.4 | 94.1 | 96.1 | 97.5 | 98.5 | 99.1 | 99.5 | 99.8 | 99.9 | 100 | 100 | 100 | 100 |
| | | **0** | **1** | **2** | **3** | **4** | **5** | **6** | **7** | **8** | **9** | **10** | **11** | **12** | **13** | **14** | **15** | **16** | **17** | **18** | **19** | **20** |
| Total | 12 | 0.1 | 8.8 | 16.7 | 24.9 | 33.1 | 41.1 | 48.8 | 55.8 | 62.0 | 67.3 | 72.0 | 76.0 | 79.4 | 82.3 | 84.9 | 87.0 | 88.8 | 90.4 | 91.8 | 92.9 | 93.9 |
| | 13 | 0.1 | 9.1 | 17.3 | 25.7 | 34.2 | 42.5 | 50.3 | 57.4 | 63.5 | 68.9 | 73.4 | 77.3 | 80.7 | 83.5 | 85.9 | 88.0 | 89.7 | 91.2 | 92.5 | 93.6 | 94.5 |
| | 14 | 0.1 | 9.4 | 17.9 | 26.6 | 35.4 | 43.9 | 51.9 | 59.0 | 65.1 | 70.4 | 74.9 | 78.7 | 82.0 | 84.7 | 87.0 | 89.0 | 90.6 | 92.0 | 93.2 | 94.2 | 95.1 |
| | 15 | 0.1 | 9.7 | 18.5 | 27.6 | 36.7 | 45.5 | 53.6 | 60.7 | 66.8 | 72.0 | 76.4 | 80.1 | 83.2 | 85.9 | 88.1 | 89.9 | 91.5 | 92.8 | 93.9 | 94.9 | 95.6 |
| | 16 | 0.1 | 10.0 | 19.2 | 28.7 | 38.1 | 47.1 | 55.3 | 62.4 | 68.5 | 73.6 | 77.9 | 81.5 | 84.5 | 87.0 | 89.1 | 90.9 | 92.3 | 93.6 | 94.6 | 95.4 | 96.2 |
| | 17 | 0.1 | 10.4 | 20.0 | 29.8 | 39.6 | 48.8 | 57.1 | 64.3 | 70.2 | 75.3 | 79.4 | 82.9 | 85.8 | 88.2 | 90.1 | 91.8 | 93.2 | 94.3 | 95.2 | 96.0 | 96.7 |
| | | **21** | **22** | **23** | **24** | **25** | **26** | **27** | **28** | **29** | **30** | **31** | **32** | **33** | **34** | **35** | **36** | **37** | **38** | **39** | **40** | |
| | 12 | 94.8 | 95.5 | 96.1 | 96.6 | 97.1 | 97.5 | 97.8 | 98.1 | 98.4 | 98.6 | 98.8 | 99.0 | 99.1 | 99.2 | 99.3 | 99.4 | 99.5 | 99.7 | 99.8 | 100 | |
| | 13 | 95.3 | 96.0 | 96.5 | 97.0 | 97.5 | 97.8 | 98.1 | 98.4 | 98.6 | 98.8 | 99.0 | 99.1 | 99.2 | 99.3 | 99.4 | 99.5 | 99.6 | 99.7 | 99.8 | 100 | |
| | 14 | 95.8 | 96.4 | 97.0 | 97.4 | 97.8 | 98.1 | 98.4 | 98.6 | 98.8 | 99.0 | 99.1 | 99.2 | 99.4 | 99.4 | 99.5 | 99.6 | 99.6 | 99.7 | 99.8 | 100 | |
| | 15 | 96.3 | 96.9 | 97.3 | 97.7 | 98.1 | 98.4 | 98.6 | 98.8 | 99.0 | 99.1 | 99.3 | 99.4 | 99.5 | 99.5 | 99.6 | 99.7 | 99.7 | 99.8 | 99.8 | 100 | |
| | 16 | 96.8 | 97.3 | 97.7 | 98.1 | 98.4 | 98.6 | 98.8 | 99.0 | 99.2 | 99.3 | 99.4 | 99.5 | 99.6 | 99.6 | 99.7 | 99.7 | 99.8 | 99.8 | 99.9 | 100 | |
| | 17 | 97.2 | 97.7 | 98.0 | 98.3 | 98.6 | 98.8 | 99.0 | 99.2 | 99.3 | 99.4 | 99.5 | 99.6 | 99.6 | 99.7 | 99.7 | 99.8 | 99.8 | 99.8 | 99.9 | 100 | |

SDQ = Strengths and Difficulties Questionnaire.

Cells containing percentiles ≥ 90 are shaded dark gray and cells containing percentiles from 80 up to 90 light gray, with the corresponding scale scores classified as falling in the 'abnormal' and 'borderline' range, respectively. The remaining scale scores are classified as 'normal'.
